# LEDitSHAKE: a lighting system to optimize the secondary metabolite content of plant cell suspension cultures

**DOI:** 10.1038/s41598-021-02762-6

**Published:** 2021-12-02

**Authors:** Ann-Katrin Beuel, Natalia Jablonka, Julia Heesel, Kevin Severin, Holger Spiegel, Stefan Rasche

**Affiliations:** 1 Fraunhofer Institute for Molecular Biology and Applied Ecology IME, Forckenbeckstraße 6, 52074 Aachen, Germany; 2 Fraunhofer Institute for Molecular Biology and Applied Ecology IME, Auf dem Aberg 1, 57392 Schmallenberg, Germany

**Keywords:** Light responses, Secondary metabolism

## Abstract

Plant secondary metabolites are widely used in the food, cosmetic and pharmaceutical industries. They can be extracted from sterile grown plant cell suspension cultures, but yields and quality strongly depend on the cultivation environment, including optimal illumination. Current shaking incubators do not allow different light wavelengths, intensities and photoperiods to be tested in parallel. We therefore developed LEDitSHAKE, a system for multiplexed customized illumination within a single shaking incubator. We used 3D printing to integrate light-emitting diode assemblies into flask housings, allowing 12 different lighting conditions (spectrum, intensity and photoperiod) to be tested simultaneously. We did a proof of principle of LEDitSHAKE using the system to optimize anthocyanin production in grapevine cell suspension cultures. The effect of 24 different light compositions on the total anthocyanin content of grapevine cell suspension cultures was determined using a Design of Experiments approach. We predicted the optimal lighting conditions for the upregulation and downregulation of 30 anthocyanins and found that short-wavelength light (blue, UV) maximized the concentration of most anthocyanins, whereas long-wavelength light (red) had the opposite effect. Therefore our results demonstrate proof of principle that the LEDitSHAKE system is suitable for the optimization of processes based on plant cell suspension cultures.

## Introduction

Plant cell suspension cultures (PCSCs) are suitable as a production system for plant secondary metabolites (PSMs), which are valuable ingredients in the food, cosmetic and pharmaceutical industries. PCSCs have several advantages over wild harvested or agriculturally grown plants as a source of PSMs. PCSCs isolate the production process from the effects of season, location or geopolitical issues, and facilitate harvesting and downstream processing^1–4^. There is also no need to use herbicides, and the sterile cultivation conditions eliminate the risk of contamination with bacterial endotoxins, further contributing to the high and consistent quality of the PSMs.

Despite the advantages described above, the growth and productivity of PCSCs is affected by many factors, including medium components, temperature, and light^3,5^. Light is not only an energy source for plants, but also triggers developmental transitions such as germination and flowering, as well as the production of PSMs^6–8^. One of the most important groups of PSMs is the anthocyanidins, and their glycosylated counterparts, the anthocyanins. These confer the red and blue coloring of plant tissues. They are attractive ingredients in the food, cosmetics and pharmaceutical industries because they act as natural colorants as well as providing antimicrobial and antioxidant activity^9,10^.

Anthocyanins accumulate in the plant cell vacuole, but the quantity and composition varies in different tissues and plant species, and also according to the environmental conditions. Under abiotic stress (e.g., UV light, high light intensities, or extreme temperatures) or biotic stress (e.g., pathogens, pest insects, or herbivores) the production of anthocyanins is upregulated and they accumulate to high levels^10^. Early studies showed that anthocyanin synthesis is light dependent^11,12^, but little is known about the effects of different wavelengths on PCSCs. This mainly reflects the absence of sophisticated technical equipment to facilitate comparative experiments. Many studies have examined the effect of light composition on plant tissue cultures (e.g., callus cultures, liquid cultures in bioreactors, and whole plants). A common limitation of these studies is the technical implementation of the experiments^13–15^. The illumination source in standard shaking incubators is typically built into the ceiling of the device, making it impossible to test different lighting conditions on several cultures simultaneously in a single incubator. Therefore, a separate incubator is needed for each light setting, which increases the cost and workload while restricting the experimental throughput.

To address the drawbacks of current platforms, we developed the LEDitSHAKE (LiS) lighting system for PCSCs in Erlenmeyer flasks, allowing the simultaneous testing of up to 12 different lighting conditions in the same shaking incubator. The influence of light composition can therefore be evaluated alone or in combination with other cultivation parameters using statistical experimental designs to reveal potential synergistic or antagonistic effects on growth and productivity. As a proof of concept, we tested a grapevine (*Vitis vinifera*) cell suspension culture, which is strongly influenced by light and accumulates high concentrations of anthocyanins^16^. We used LiS to determine, for the first time, the effect of different wavelengths of light and mixtures thereof on the production of PSMs by cell suspension cultures within the same shaking incubator.

## Results

### LEDitSHAKE

**Figure 1:**
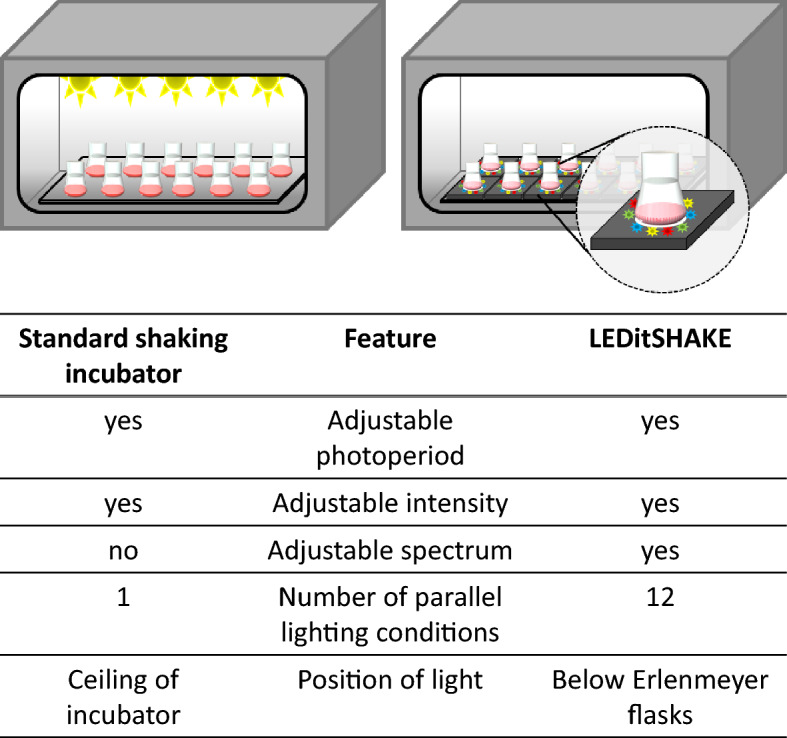
The key features of the LEDitSHAKE (LiS) system compared to a state-of-the-art illuminated shaking incubator. Schematic drawings of both setups are included. LiS is shown with the lightproof cases removed so that the light source can be seen.

**Figure 2:**
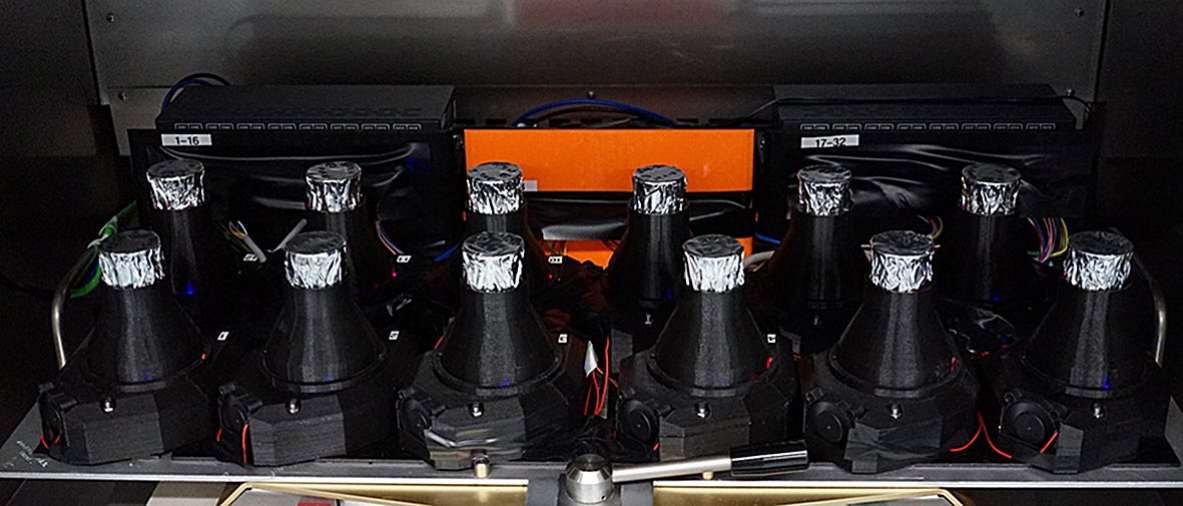
The LiS system equipped with 12 Erlenmeyer flasks in a shaking incubator. The 12 individual LiS vessels are shown in the foreground. The three boxes in the background contain dimmer controls connected to the LEDs and software.

The patented LiS system^17^ is compared to a standard shaking incubator in Fig. 1. It allows 12 different lighting conditions to be tested in parallel within one shaking incubator (Fig. 2).

To develop the LiS system, we integrated light-emitting diode (LED) assemblies into a custom designed, 3D printed flask housing for 250-mL Erlenmeyer flasks. Adapters for 50-mL and 100-mL flasks were also printed. Six individual OSRAM LEDs (hyperred 660 nm, green 528 nm, blue 455 nm, far red 730 nm, UV 365 nm and white 420–710 nm) were positioned beneath the flasks as light sources so that the light intensity and wavelength composition could be adjusted individually for each flask according to experimental requirements. Each LED was connected to a digital multiplex (DMX) decoder and controlled using the E: CUE lighting application suite. We calibrated each LED in the LiS to ensure that the light intensity reached the levels needed for the experiments. We also printed a lightproof case to secure each flask and prevent the scattering of light between flasks, which is important when different lighting conditions are tested in parallel. A ventilation system was also included to dissipate the heat generated by the LEDs. To confirm that our lighting system was suitable for optimization experiments, we used a grapevine (*Vitis vinifera*) cell suspension culture to investigate how the anthocyanin content and composition is influenced by light. Therefore we used two LiS systems at the same time, so that 24 different lighting conditions could be tested in parallel.

### Effect of light on the anthocyanin content and composition of the cell suspension cultures

Having established the principle of multiplexed customized illumination within a single incubator, we investigated the effects of different lighting conditions on the anthocyanin content and composition of grapevine cell suspension cultures to confirm that the LiS system works in practice. We used two LiS systems to test 24 lighting conditions in parallel, to determine the effect of red, green, blue and UV light and their mixtures. A Design of Experiments (DoE) approach (IV-optimal response surface method, RSM) was used to cover the design space, with red, green, blue and UV light as the four factors, resulting in 24 runs (Table 1). The factor ranges, types and levels of the DoE setup are shown in Table 2.

**Table 1:** Light compositions to determine the effect of light on the anthocyanin content of grapevine cultures.

No	Intensity red light [µmol m^−2^ s^−1^] 6 am – 10 pm	Intensity green light [µmol m^−2^ s^−1^] 6 am – 10 pm	Intensity blue light [µmol m^−2^ s^−1^] 6 am – 10 pm	UV light [9 µmol m^−2^ s^−1^] 12 pm – 1 pm
1	50	0	0	On
2	0	25	25	On
3	0	50	0	Off
4	8.3	8.3	33.3	Off
5	8.3	33.3	8.3	On
6	0	0	50	Off
7	25	25	0	Off
8	25	25	0	On
9	0	0	50	On
10	50	0	0	Off
11	0	50	0	On
12	0	25	25	Off
13	16.6	16.6	16.6	Off
14	0	25	25	On
15	16.6	16.6	16.6	On
16	8.3	33.3	8.3	Off
17	25	0	25	On
18	25	25	0	Off
19	33.3	8.3	8.3	Off
20	8.3	8.3	33.3	On
21	25	0	25	Off
22	25	0	25	On
23	25	25	0	On
24	25	0	25	Off

Generated using Design-Expert v11.0.3.

**Table 2:** Overview of mixture components, factors and factor levels used in the response surface model.

Component/factor	Name	Unit	Type	Min	Max	L1	L2	L3	L4
A	Red	µmol m^−2^ s^−1^	Mixture	0	50	–	–	–	–
B	Green	µmol m^−2^ s^−1^	Mixture	0	50	–	–	–	–
C	Blue	µmol m^−2^ s^−1^	Mixture	0	50	–	–	–	–
D	Week	–	Discrete	–	–	1	2	3	4
E	UV	µmol m^−2^ s^−1^	Nominal	–	–	On	Off	–	–

To plan the DoE-setup we used a mixture design with the intensity of red, green and blue light as the three components of the mixture (component A-C). The sum of all three components had to be 50 µmol m^−2^ s^−1^ leading to a minimum intensity (Min.) of 0 µmol m^−2^ s^−1^ and a maximum (Max.) of 50 µmol m^−2^ s^−1^ for each component itself. We included the cultivation week as a discrete factor (factor D) with four levels (L1 = Level 1, L2 = Level 2, L3 = Level 3, L4 = Level 4) where each level represents one week of cultivation. The intensity of UV light (factor E) was included as a nominal factor with two levels (L1 = Level 1, L2 = Level 2), where L1 means UV light was turned on and L2 means that UV light was turned off. These components and factors with their levels were used to generate the DoE-runs shown in Table 1.

We prepared 26 replicate grapevine suspension cultures from the same source culture (standard shaking incubator, light positioned in the ceiling, 16-h photoperiod, 80 µmol m^−2^ s^−1^) to ensure equivalent starting conditions, 24 of which were cultivated in the LiS system according to the RSM plan. The remaining two cultures were used as controls, one cultivated under white light (standard shaking incubator, light positioned in the ceiling, 16-h photoperiod, 80 µmol m^−2^ s^−1^), which are the same conditions as the source culture, and the other in darkness. All cultures were cultivated for 4 weeks with weekly subculturing and sampling. The growth of the cultures was determined by measuring the packed cell volume (PCV) weekly before subculturing. Statistical analysis of the PCV data (analysis of variance, ANOVA) revealed that the lighting conditions did not significantly affect the growth of the cultures (*p* = 0.797). Accordingly, we focused solely on the anthocyanin content of the cultures for subsequent analysis.

We measured the concentrations of the most important anthocyanidins, namely cyanidin, delphinidin, malvidin, peonidin, petunidin and pelargonidin^18,19^, by liquid chromatography-ion mobility separation-high resolution mass spectrometry (LC-IMS-HRMS). We also measured the corresponding anthocyanins, the glycoside derivatives of these aglycone precursors (specifically the glucosides, di-glucosides, acetylglucosides and coumaroylglucosides). The concentrations of each anthocyanin and the total anthocyanin content were then plotted against the cultivation time and lighting conditions, and analyzed using the Design-Expert software (Design-Expert® software, version 11.0.3, Stat-Ease, Inc., Minneapolis, MN, USA, www.statease.com). The total anthocyanin content is shown in Fig. 3, and the individual anthocyanins are shown in Supplementary Figs. 1–36.

**Figure 3:**
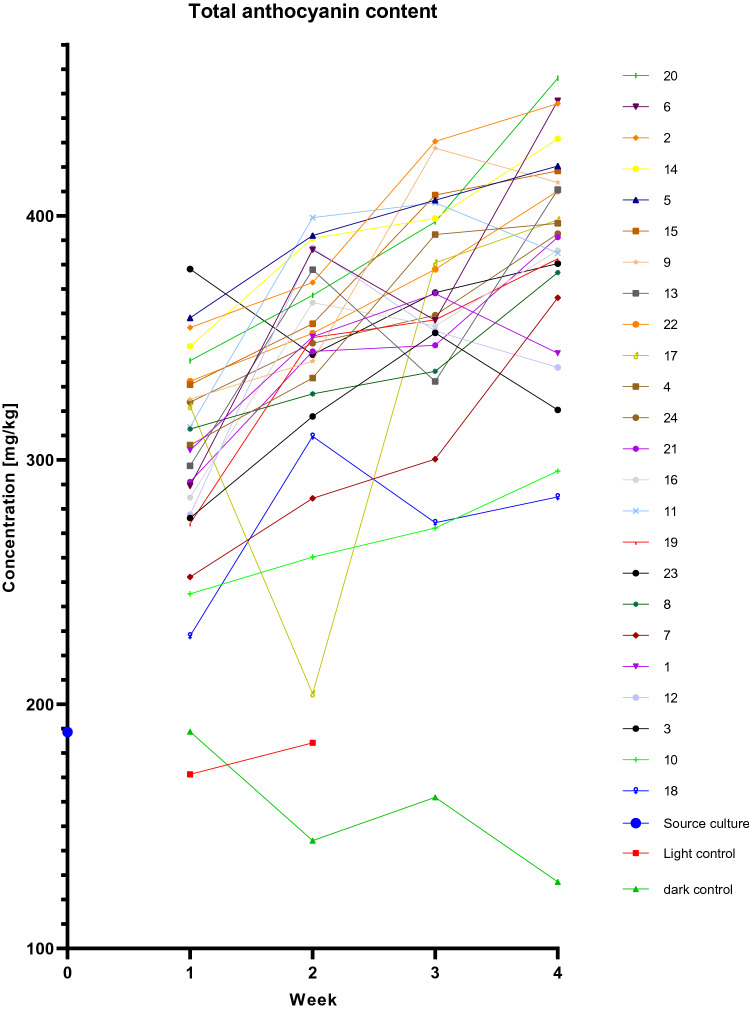
Total anthocyanin content of grapevine cultures under different lighting conditions and at different time points. All cultures originated from the same source culture. All 26 replicate cultures were cultivated for 4 weeks at 26 °C, shaking at 140 rpm. Every week, 15% of the cells (v/v) were subcultured and the remaining cells were collected for LC-IMS-HRMS analysis to determine anthocyanin concentrations. Each line represents a lighting condition: conditions 1–24 are based on the DoE model (Table 1) with an intensity of red + green + blue light = 50 µmol m^2^ s ^1^ and UV light either off or on for 1 h (12–1 pm) each day (9 µmol m^2^ s^1^). Those cultures were cultivated in the LiS under 24 different lighting conditions with a 16-h photoperiod. The light control was cultivated according to the conditions of the routine/source culture (standard shaking incubator, light positioned in the ceiling, 16-h photoperiod, 80 µmol m^2^ s^1^), but was contaminated after 3 weeks, therefore the data represent only 2 weeks of cultivation. The dark control was cultivated in the absence of light. There seemed to be an error with sample preparation or analysis of culture 17 in week 2, but we decided to keep the point in the graph in order to not delete any data.

Figure 3 shows the total anthocyanin concentrations of all samples tested in weeks 1 to 4. The source culture had an anthocyanin concentration of 188.6 mg/kg in week 0. All samples from the LiS system accumulated more anthocyanins than the source culture, whereas the anthocyanin content of the dark control decreased to 127.3 mg/kg after 4 weeks. In the LiS system, we found that cultivation condition 20 (8.3 µmol m^−2^ s^−1^ red, 8.3 µmol m^−2^ s^−1^ green, 33.3 µmol m^−2^ s^−1^ blue, UV on) led to an anthocyanin concentration of 456.4 mg/kg after 4 weeks, which was 2.42-fold higher than the source culture. Nine conditions increased the concentration by 2.15-fold or more after 4 weeks. Additionally, we found that condition 23 (25 µmol m^−2^ s^−1^ red, 25 µmol m^−2^ s^−1^ green, 0 µmol m^−2^ s^−1^ blue, UV on) doubled the anthocyanin concentration after only 1 week of cultivation. Therefore we decided to include the cultivation period (“week”) as a numeric factor in the Design-Expert software using “historical data”. The original factors, ranges and levels for the model stayed the same (24 runs = 24 lighting conditions) but the cultivation period was included with 4 levels (week 1, week 2, week 3 and week 4). This resulted in 4 × 24 = 96 runs. The data we had already acquired was then used to generate response surface models and corresponding ANOVA for each anthocyanin (Table 3). In all but four of the models we found significant correlations and the response surface graphs as well as the ANOVA showed that each anthocyanin was differently affected by light. The remaining four models (Mv, Mv-DiGlu, Pg-DiGlu and Pt-DiGlu) could not be analyzed because the anthocyanin concentration was zero in every sample. However, the concentration of most anthocyanins increased under short-wave UV and blue light but decreased under long-wave red light.

**Table 3:** Factors and factor interactions used to predict anthocyanin concentrations and model characteristics to ensure significance.

Model	F-value	*p*-value	Significant factors	Lack of fit	R^2^	Adjusted R^2^	Predicted R^2^
Cy	13.04	< 0.0001	A, B, C, AC, AE, CD, ACE	0.7225	0.509	0.4701	0.4385
Cy-Glu	42.47	< 0.0001	A, B, C, AC, AD, AE, BC, BD, BE, CD, CE, ACE	0.596	0.8491	0.8291	0.801
Cy-DiGlu	23.53	< 0.0001	A, B, C, AD, AE, BD, BE, CD, CE, ADE	0.5744	0.7112	0.6809	0.6411
Cy-AcGlu	65.48	< 0.0001	A, B, C, AD, AE, BD, BE, CD	0.8519	0.8389	0.8261	0.8086
Cy-CouGlu	14.15	< 0.0001	A, B, C, AE, BE, CD, ABC	1.7	0.6495	0.6036	0.546
Dp	40.99	< 0.0001	A, B, C, AC, AE, BE, CE, ACE	1.11	0.9474	0.9243	0.8968
Dp-Glu	38.37	< 0.0001	A, B, C, AE, BE, CE, ABC, ACE	0.4955	0.8187	0.7973	0.7687
Dp-DiGlu	66.06	< 0.0001	A, B, C, AC, AD, AE, BD, BE, CD, CE, ACE, BDE, BD^2^, CD^2^, ACDE	0.9328	0.9351	0.9209	0.9046
Dp-AcGlu	7.95	< 0.0001	B, C, AB, BE, CD, CE, ABC, ABE, BCD, BD^2^, ABCE, BCDE, ABD^2^, BCD^2^, BD^2^E, CD^3^, ABD^2^E, BCD^2^E	0.9472	0.8291	0.7249	0.4681
Dp-CouGlu	42.34	< 0.0001	A, B, C, AE, BE, CD, CE, BD^2^, CD^2^	0.7929	0.8328	0.8131	0.793
Mv	NA	NA	NA	NA	NA	NA	NA
Mv-Glu	21.31	< 0.0001	A, B, C, AE, BE, CD, CE, BD^2^	0.594	0.7362	0.7017	0.6633
Mv-DiGlu	NA	NA	NA	NA	NA	NA	NA
Mv-AcGlu	12.49	< 0.0001	A, B, C, AD, AE, BD, CE, CDE, BD^2^, CD^2^, AD^2^E, CD^2^E, CD	1.01	0.7449	0.6852	0.5661
Mv-CouGlu	24.15	< 0.0001	A, B, C, AB, AE, BE, CE, ABE, ACE, BDE, AD^2^, BD^2^, CD^2^, AD^2^E, BD^2^E, AD^3^, BD^3^, CD^3^	0.5172	0.901	0.8637	0.7991
Pg	13.62	< 0.0001	D, E, DE	1.76	0.3075	0.2849	0.2464
Pg-Glu	47.46	< 0.0001	A, B, C, AC, AD, AE, BD, BE, CD, CE	0.6987	0.8481	0.8302	0.8034
Pg-DiGlu	NA	NA	NA	NA	NA	NA	NA
Pg-AcGlu	17.6	< 0.0001	B, C, BE, CD	1.14	0.4943	0.4662	0.4334
Pg-CouGlu	22.41	< 0.0001	A, B, C, AD, AE, BD, BE, CD, CE, AD^2^, BD^2^, CD^2^	0.6679	0.7459	0.7126	0.6635
Pn	21.55	< 0.0001	A, B, C, AC, AD, CD	0.8591	0.5449	0.5196	0.4862
Pn-Glu	30.44	< 0.0001	A, B, C, AC, AD, AE, BD, BE, CD, CE, ABC, ACE, AD^2^, BD^2^	0.8322	0.8705	0.8419	0.7999
Pn-DiGlu	74.55	< 0.0001	D, E, DE	0.6797	0.7085	0.699	0,6832
Pn-AcGlu	73.67	< 0.0001	A, B, C, AD, AE, BD, BE, CD, BD^2^	0.9864	0.8714	0.8595	0.8457
Pn-CouGlu	12.21	< 0.0001	A, B, C, AB, BD, CD, ABC, BCE, BD^2^, ABCE, ABD^2^	0.7381	0.765	0.7024	0.619
Pt	6.2	< 0.0001	AC, ACD^2^	0.4031	0.3934	0.33	0.2405
Pt-Glu	114.43	< 0.0001	A, B, C, AB, AC, AE, BD, BE, CD, CE, ABC, ACE	1.57	0.9436	0.9354	0.927
Pt-DiGlu	NA	NA	NA	NA	NA	NA	NA
Pt-AcGlu	21.52	< 0.0001	B, C, BD, BE, CD, CE, BDE, CDE, BD^2^, BD^2^E	0.5115	0.7168	0.6835	0.6447
Pt-CouGlu	28.77	< 0.0001	A, B, AD, AE, BD, CD, ACE, BCE	1.32	0.8202	0.7917	0.7655

We preselected factors showing a significant influence on the biomass yield by automated backward selection with a p-value threshold of 0.100. Factors with a p-value > 0.1 were removed, except those needed to maintain the model hierarchy. A p-value of 0.1 indicates a significance (α) level of 10%. Cy = Cyanidin, Dp = Delphinidin, Mv = Malvidin, Pg = Pelargonidin, Pn = Peonidin, Pt = Petunidin, Glu = Glucoside, DiGlu = Diglucoside, AcGlu = Acetylglucoside, CouGlu = Coumaroylglucoside. Factors: A = Red, B = Green, C = Blue, D = Week, E = UV.

**Figure 4:**
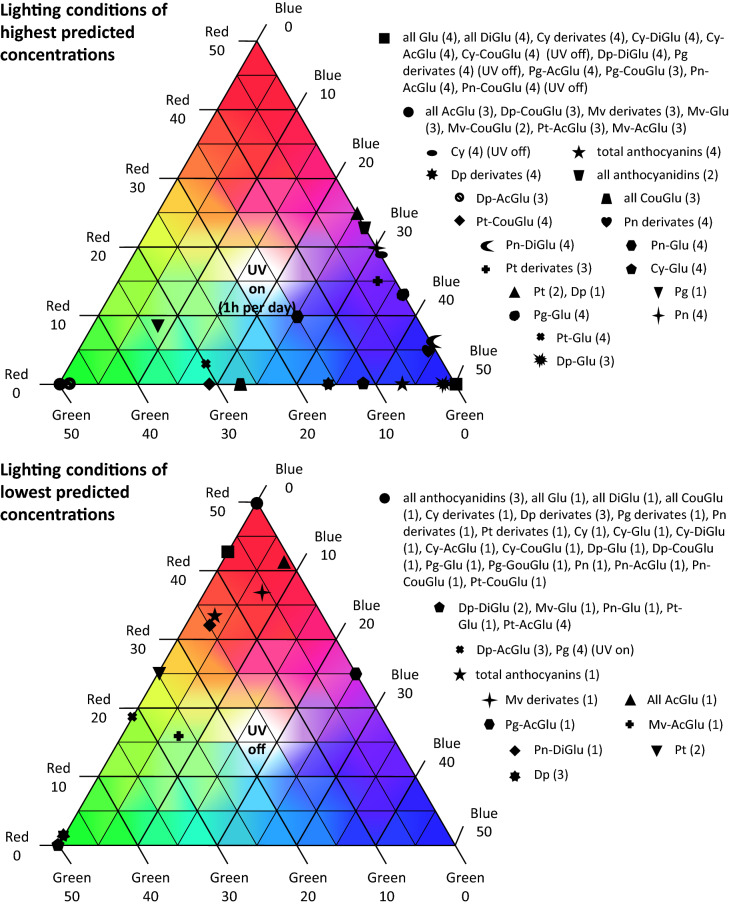
Optimal lighting conditions predicted to achieve the highest and lowest concentrations of each anthocyanin. Lighting conditions and concentrations were predicted using the Design-Expert tool “numerical optimization”, in which we chose a target (= anthocyanin or groups of anthocyanins) to maximize and minimize. Values were calculated based upon the previously generated RSMs. The color triangle represents the design space for the mixture design (red, green and blue), so every point on the triangle is a potential lighting condition within the ranges of the design. The numbers after the color (e.g. Red 50) specify the intensity of the light in µmol m^−2^ s^−1^. Every point marked with a symbol represents the optimal lighting condition for the anthocyanin annotated with the same symbol. The number in brackets after the name of the anthocyanin is the cultivation time. “UV on/off” after the bracket means that for this anthocyanin the predicted UV level is different from the information within the triangle. The upper triangle shows the lighting conditions for upregulation and the triangle below shows the lighting conditions for downregulation.

Based on these results from cultures 1 to 24 in all four weeks of the actual experiment, we used Design-Expert to predict the lighting conditions that should yield the highest and lowest concentrations of each anthocyanin, as well as groups of anthocyanins and total anthocyanins. We picked the most suitable solution from those suggested by the software and plotted the resulting lighting conditions onto a color triangle which represents the design space (Fig. 4).

Different lighting conditions are needed to upregulate and downregulate the anthocyanin content. For upregulation, 34 of 38 target anthocyanins reached their maximum concentrations when the UV light was turned on, whereas downregulation was achieved when the UV light was turned off for all but one target. When comparing the lighting conditions for upregulation and downregulation, we found that short-wavelength light (blue 455 nm, UV 365 nm) promotes the accumulation of anthocyanins whereas long-wavelength light (red 660 nm) has the opposite effect. The effect of green light (528 nm) was dependent on the specific anthocyanin. For example, the highest concentration of Pn-Glu was predicted to occur following exposure to 10 µmol m^−2^ s^−1^ red, 15 µmol m^−2^ s^−1^ green and 25 µmol m^−2^ s^−1^ blue with UV turned on for 1 h/day. Based on our calculations, the optimal lighting conditions would result in a Pn-Glu concentration of 143.6 mg/kg after 4 weeks, which is a 2.6-fold increase compared to the source culture of this experiment (Pn-Glu concentration 55.3 mg/kg). Two examples showing the best lighting conditions for upregulation of anthocyanin concentrations and two showing the best lighting conditions for downregulation are provided in Table 4.

**Table 4:** Predicted light compositions to achieve the highest respectively lowest concentrations of four anthocyanins.

Goal	Intensity red light [µmol m^−2^ s^−1^]	Intensity green light [µmol m^−2^ s^−1^]	Intensity blue light [µmol m^−2^ s^−1^]	UV
Pn-Glu up	10	15	25	on
Cy-Glu up	0	12	38	on
Dp-Glu down	50	0	0	off
Pt-Glu down	0	50	0	off

Values and light compositions were calculated using the numerical optimization tool of the Design-Expert software and were compared to the source culture of the main experiment. Conc. = Concentration, Cy = Cyanidin, Dp = Delphinidin, Pn = Peonidin, Pt = Petunidin, Glu = Glucoside, Up = Upregulation (the goal is to maximize the concentration), Down = Downregulation (the goal is to minimize the concentration).

**Figure 5:**
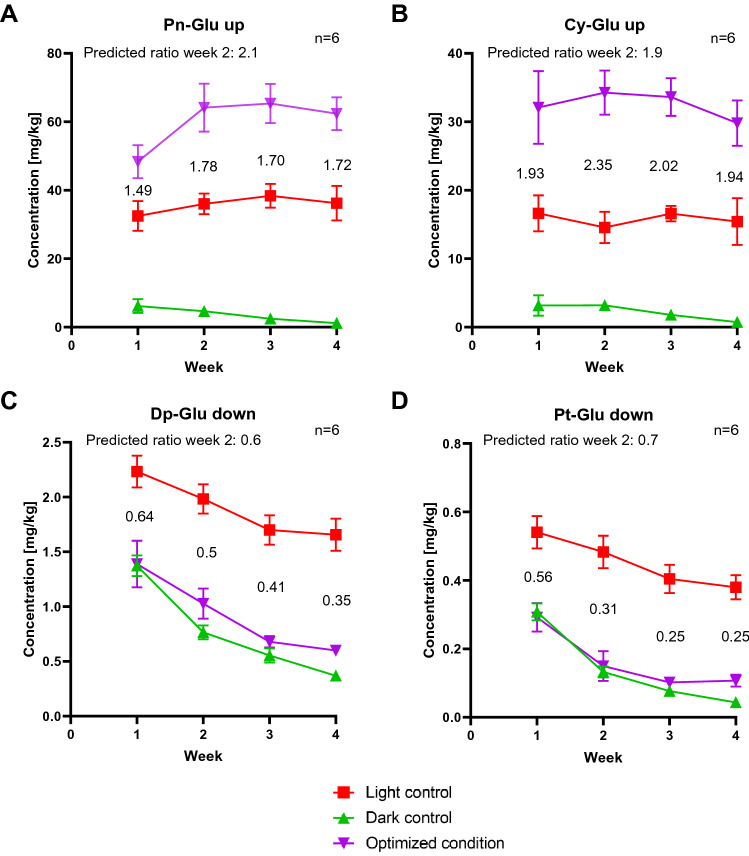
Concentration of four different anthocyanins of grapevine cultures under different lighting conditions during 4 weeks. All cultures originated from the same source culture. All 36 replicate cultures were cultivated for 4 weeks at 26 °C, shaking at 140 rpm. Every week, 15% of the cells (v/v) were subcultured and the remaining cells were collected for LC-IMS-HRMS analysis to determine anthocyanin concentrations. Six cultures per anthocyanin target (see Table 4) were grown under their optimized lighting condition (purple triangle) for upregulation (up) or downregulation (down). Those cultures were cultivated in the LiS under their optimized lighting conditions with a 16-h photoperiod. The six light controls (red square) were cultivated according to the conditions of the routine/source culture (standard shaking incubator, light positioned in the ceiling, 16-h photoperiod, 80 µmol m^2^ s^1^), the six dark controls were cultivated in the absence of light (green triangle). The ratio between the anthocyanin concentrations in the cultures under optimized lighting conditions and the anthocyanin concentrations in the cultures under standard lighting conditions were calculated and are shown in the graphs for every week, as well as the predicted ratio based on the calculations from the main experiment are shown for week 2. Each graph represents a different anthocyanin: (**A**) Peonidin-Glucoside (Pn-Glu), (**B**) Cyanidin-Glucoside (Cy-Glu), (**C**) Delphinidin-Glucoside (Dp-Glu) and (**D**) Petunidin-Glucoside (Pt-Glu).

The predicted lighting conditions from Table 4 were used as a control experiment for our model. We decided to use 6 replicates per lighting conditions as well as 6 light and 6 dark controls, resulting in 36 replicate grapevine suspension cultures that were needed for the control experiment. To ensure equivalent starting conditions, those 36 replicates were prepared from the same source culture (standard shaking incubator, light positioned in the ceiling, 16-h photoperiod, 80 µmol m^−2^ s^−1^). 24 cultures were cultivated in the LiS system with lighting conditions according to Table 4 with 6 replicates each, the 6 light controls were cultivated under white light (standard shaking incubator, light positioned in the ceiling, 16-h photoperiod, 80 µmol m^−2^ s^−1^), which are the same conditions than the source culture, and the 6 remaining cultures in darkness. All cultures were cultivated for 4 weeks with weekly subculturing and sampling. The concentrations of the target anthocyanins (Pn-Glu, Dp-Glu, Cy-Glu and Pt-Glu) in the cultures cultivated under their optimized lighting conditions and under control conditions were analyzed via LC-IMS-HRMS as in the main experiment and are shown in Fig. 5.

Figure 5 shows the concentrations of the target anthocyanins with the aim of upregulation (up) or downregulation (down) (A: Pn-Glu up, B: Cy-Glu up, C: Dp-Glu down and D: Pt-Glu down) in the cultures cultivated under the optimized lighting conditions predicted to be required to reach the respective aims and under control conditions in weeks 1 to 4 as well as the corresponding concentration in the source culture in week 0. For better comparison with the main experiment we focus on week 2, as this was the last week with data for the light control in the main experiment due to contamination. Therefore we calculated the ratio between the anthocyanin concentrations in the cultures under optimized lighting conditions in week 2 and the anthocyanin concentrations in the cultures under standard lighting conditions in week 2. We then compared the ratios of the main experiment (predicted by the DoE program) with the actual ratios of the control experiment. The ratios between the cultivation conditions for the other weeks are also shown in Fig. 5.

The average Pn-Glu concentration was 64.2 mg/kg in week 2 when cultivated under the optimized lighting conditions (10 µmol m^−2^ s^−1^ red, 15 µmol m^−2^ s^−1^ green, 25 µmol m^−2^ s^−1^ blue and UV turned on). The average Pn-Glu concentration in week 2 was 36.0 mg/kg in the light controls and 4.7 mg/kg in the dark controls. The ratio between the optimized condition and the light control therefore equals 1.8, meaning 80% more Pn-Glu was accumulated when the cells were cultivated under optimized lighting conditions. Significant differences between the cultures regarding the Pn-Glu concentrations were also confirmed by ANOVA and Tukey’s multiple comparison test.

The highest average Cy-Glu concentration in week 2 was found in the cultures cultivated under the optimized lighting conditions (12 µmol m^−2^ s^−1^ green, 38 µmol m^−2^ s^−1^ blue and UV turned on) with 34.3 mg/kg, whereas the average concentration in the light controls in week 2 was 14.6 mg/kg and in the dark controls 3.2 mg/kg. This means 130% more Cy-Glu was found in cells cultivated under optimized conditions than under standard conditions (ratio 2.3). ANOVA and Tukey’s multiple comparison test confirmed significant differences between the applied lighting conditions and the resulting Cy-Glu concentrations.

In the cultures cultivated under optimized conditions for Dp-Glu downregulation (50 µmol m^−2^ s^−1^ red and UV turned off) the average Dp-Glu concentration was 1.0 mg/kg, whereas the average concentration in the light controls was 2.0 mg/kg while the average concentration in the dark controls was 0.8 mg/kg. Therefore the resulting ratio between the optimized condition and the standard condition is 0.5, 50% less Dp-Glu was accumulated. In week 1 there was no significant correlation between the dark control and the optimized condition, but in all other weeks significant differences between all three lighting conditions were found by ANOVA and Tukey’s multiple comparison test.

The average Pt-Glu concentration was 0.5 mg/kg in light controls in week 2 and 0.13 mg/kg in dark controls. In the cultures cultivated under optimized conditions for Pt-Glu downregulation (50 µmol m^−2^ s^−1^ green and UV turned off) the average concentration in week 2 was 0.15 mg/kg, meaning 70% less Pt-Glu was found under optimized conditions (ratio 0.3). Significant differences between the light control and the optimized condition respectively between the light and the dark control were confirmed by ANOVA and Tukey’s multiple comparison test in all weeks. In week 3 and 4 significant differences were also found between optimized conditions and the dark controls, which was not the case in weeks 1 and 2. All *p*-values for the control experiment can be found in Table 5.

**Table 5:** *p*-values of Tukey’s multiple comparison tests for the comparison of concentrations of the four target anthocyanins from cultures cultivated under different lighting conditions.

Target anthocyanin	Week	Comparison	*p*-value	Significant
Pn-Glu	1	Light control vs. Dark control	< 0,0001	Yes
		Light control vs. Optimized condition	0,0004	Yes
		Dark control vs. Optimized condition	< 0,0001	Yes
	2	Light control vs. Dark control	< 0,0001	Yes
		Light control vs. Optimized condition	0,0001	Yes
		Dark control vs. Optimized condition	< 0,0001	Yes
	3	Light control vs. Dark control	< 0,0001	Yes
		Light control vs. Optimized condition	< 0,0001	Yes
		Dark control vs. Optimized condition	< 0,0001	Yes
	4	Light control vs. Dark control	< 0,0001	Yes
		Light control vs. Optimized condition	< 0,0001	Yes
		Dark control vs. Optimized condition	< 0,0001	Yes
Cy-Glu	1	Light control vs. Dark control	< 0,0001	Yes
		Light control vs. Optimized condition	0,0126	Yes
		Dark control vs. Optimized condition	0,0024	Yes
	2	Light control vs. Dark control	< 0,0001	Yes
		Light control vs. Optimized condition	0,0003	Yes
		Dark control vs. Optimized condition	0,0006	Yes
	3	Light control vs. Dark control	< 0,0001	Yes
		Light control vs. Optimized condition	0,0011	Yes
		Dark control vs. Optimized condition	0,0003	Yes
	4	Light control vs. Dark control	0,0003	Yes
		Light control vs. Optimized condition	0,0009	Yes
		Dark control vs. Optimized condition	0,0009	Yes
Dp-Glu	1	Light control vs. Dark control	< 0,0001	Yes
		Light control vs. Optimized condition	< 0,0001	Yes
		Dark control vs. Optimized condition	0,9838	No
	2	Light control vs. Dark control	< 0,0001	Yes
		Light control vs. Optimized condition	< 0,0001	Yes
		Dark control vs. Optimized condition	0,0091	Yes
	3	Light control vs. Dark control	< 0,0001	Yes
		Light control vs. Optimized condition	< 0,0001	Yes
		Dark control vs. Optimized condition	0,0109	Yes
	4	Light control vs. Dark control	< 0,0001	Yes
		Light control vs. Optimized condition	< 0,0001	Yes
		Dark control vs. Optimized condition	< 0,0001	Yes
Pt-Glu	1	Light control vs. Dark control	< 0,0001	Yes
		Light control vs. Optimized condition	< 0,0001	Yes
		Dark control vs. Optimized condition	0,7105	No
	2	Light control vs. Dark control	< 0,0001	Yes
		Light control vs. Optimized condition	< 0,0001	Yes
		Dark control vs. Optimized condition	0,6488	No
	3	Light control vs. Dark control	< 0,0001	Yes
		Light control vs. Optimized condition	< 0,0001	Yes
		Dark control vs. Optimized condition	0,0081	Yes
	4	Light control vs. Dark control	< 0,0001	Yes
		Light control vs. Optimized condition	< 0,0001	Yes
		Dark control vs. Optimized condition	0,0003	Yes

Tukey’s multiple comparison test was done using GraphPad Prism v8.4.3 (GraphPad Software, San Diego, CA, USA). A significance (α) level of 5% was used.

The other predicted lighting conditions from the DoE can be used as the basis for further control experiments and all lighting conditions can be applied for further optimization experiments.

## Discussion and conclusions

Experimental analysis of optimal lighting conditions for PCSCs is currently limited by the design of shaking incubators. All commercially available systems for the illumination of cell cultures provide white light with the light source positioned in the ceiling of the incubator, ruling out experiments testing different light spectra, intensities and photoperiods simultaneously. Moreover, some shaking incubators are equipped with fluorescent tubes while others are equipped with white LED panels. Many studies concerning the effect of light on plant or plant cell cultivation in vitro do not specify the type of light source, making it difficult to compare results between studies^13^. Furthermore the most widely-used fluorescent tubes have no defined spectrum, and the light intensity decreases over time. We recommend LEDs as light sources for studies of secondary metabolism in plants or plant cells cultivated in vitro because LEDs allow the lighting conditions to be controlled more precisely. This trend can already be seen in horticultural lighting, where the use of LED systems for research has increased over the last few years^20,21^. A growing demand for LEDs is therefore anticipated for PCSCs^13^.

Recently, 3D printing has been used to develop modular vessels including LED lighting for plant tissue culture^14^, allowing the uniform analysis of explants under different light conditions. The parallel testing of multiple lighting conditions for PCSCs has previously been reported using a custom lighting rig consisting of six compartments^22^. LEDs of the same wavelength were installed at the top of each compartment but did not provide the ability to mix wavelengths or test different intensities and photoperiods simultaneously.

To address this issue, we have developed LiS, the first modular lighting system for PCSCs, which allows the multiplex cultivation of PCSCs under different lighting conditions within a single shaking incubator. For the first time, this also allows the inclusion of lighting parameters in the statistical design approach known as DoE, which finds interactions between factors affecting cultivation performance and has the ability to predict responses, thereby offering a holistic view of the cultivation process^5,23,24^.

The LiS was constructed by using 3D printing to generate custom light-proof flask housings that can be equipped with specialized LEDs, allowing us to implement, adjust and improve the design using simple and inexpensive tools and methods that are suitable for on-site applications. The final setup allows customized illumination to be applied to 12 individual Erlenmeyer flasks by positioning LEDs below each flask, each within the confines of a light-proof casing. The LEDs in each module are easy to replace, allowing user-defined light wavelengths and intensities to be tested in future experiments. We can also use our software to calibrate the LEDs. This ensures that every LED reaches the intensity needed for the experiment, and also allows recalibration when LEDs are replaced or when more LEDs of the same wavelength are used under one flask to achieve higher intensities. LiS offers the ability to match LED wavelengths and light intensities to plant photoreceptors and to the needs of different plant species. The ability to select specific wavelengths using LEDs rather than fluorescent lamps is one of the main advantages of LEDs. Others include the long operational life, the low power consumption and the small size^7^. Furthermore, 3D printing offers flexibility in terms of the cultivation vessel. The LiS used in this study was designed for 250-mL Erlenmeyer flasks, but we also printed adapters for 50-mL and 100-mL flasks and are currently working on a system for Optimum Growth 5-L flasks so that small-scale screening for optimal conditions can be followed by scale-up experiments with PCSCs in photo-bioreactors. The LiS accelerates this process because less culture is needed for screening and 12 lighting conditions can be tested simultaneously instead of serially in a standard shaking incubator. The concept used to develop and produce LiS was also applied to produce a light screening system for callus cultures and for lab-scale bioreactors and consist of the same LEDs and control unit. With those systems the optimized lighting conditions optimized from LiS-experiments could also be transferred to bioreactors. Moreover the screening and optimization of lighting conditions could already start at the callus stage of plant cell cultures.

It was important to confirm the practical application and robustness of the LiS system, so we investigated the effect of different lighting conditions on the production of anthocyanins using grapevine PCSCs, which are highly sensitive to light^16,25^. We therefore set up a DoE to investigate the anthocyanin content of cultures illuminated with different combinations of red, green, blue and UV light, and additionally cultivated cells under white light (= standard condition) and in darkness in standard shaking incubators as controls. All cultures were replicates of the source culture cultivated under standard lighting conditions, which means white light.

PCV data showed that the growth of the grapevine cultures was not significantly affected by the lighting conditions, confirming that LiS is suitable for PCSCs. This was supported by the simulation of standard lighting conditions (16-h photoperiod, white light below the flasks, 80 µmol m^−2^ s^−1^) in the LiS and the comparison of PCV data to cultures in a standard shaking incubator (16-h photoperiod, white light above the flasks, 80 µmol m^−2^ s^−1^). The position of the light source (above the flasks in the standard shaking incubator vs. below the flask in LiS) did not affect the growth of the cultures, which was similar in both devices (data not shown). However, we identified a PCSC that is typically cultivated in the absence of light, but where a specific lighting condition increased its growth rate (manuscript in preparation).

We compared the total anthocyanin content of the grapevine source culture and the cells cultivated in the LiS, and found a condition (8.3 µmol m^−2^ s^−1^ red, 8.3 µmol m^−2^ s^−1^ green, 33.3 µmol m^−2^ s^−1^ blue, UV on) that achieved a 2.42-fold increase in the total anthocyanin concentration. Furthermore, our predictions showed that UV light was required to achieve the highest yield of all but four anthocyanin targets, whereas the absence of UV light was required for the depletion of all but one of the anthocyanins. This observation probably reflects the natural role of anthocyanins in the protection of plants against UV light^10^.

Although we anticipated the modulation of anthocyanin levels by light, our experiments revealed that every anthocyanin reacted differently to specific wavelengths, resulting in complex profiles of anthocyanin accumulation and/or depletion depending on the lighting conditions. We found that green light can upregulate the concentration of one anthocyanin while downregulating the concentration of another, so based on our predictions it seems possible to increase or decrease the accumulation of specific anthocyanins by applying the optimal lighting conditions.

The predicted optimal lighting conditions for the upregulation and downregulation of four target anthocyanins were then used for a control experiment in the LiS. The DoE software predicted the best lighting conditions for all anthocyanins based on the concentrations that were measured in all four weeks of cultivation. As DoE-approaches are normally done without control cultures, our additionally cultivated light control, which was contaminated after week 2, did not affect the DoE prediction and data from all four weeks of cultures 1 to 24 could be used in order to get the best possible prediction. For culture 17 in week 2 there seemed to be an error with sample preparation or analysis. We kept the data points in Fig. 3 and the additional file in order not to delete any data. However, the DoE-software marked the anthocyanin concentrations of culture 17 in week 2 as possible deviations/outliers and therefore we decided to exclude these points for the calculation of the optimal lighting conditions.

For each target anthocyanin six replicate suspension cultures were prepared and cultivated under the predicted optimal lighting condition. Additionally, six light controls (= standard condition) and six dark controls were cultivated. The aim was to upregulate two anthocyanins (Pn-Glu and Cy-Glu) and to downregulate two other anthocyanins (Dp-Glu and Pt-Glu) when comparing the concentration of those anthocyanins between the cells cultivated under optimized lighting conditions with the ones cultivated under standard conditions (white light). For easier comparison of the main and the control experiment we decided to calculate the ratio between the anthocyanin concentrations under those two conditions (optimized lighting condition vs. standard white light) instead of looking at absolute values, because the absolute values even under standard lighting conditions vary over time for example due to biological variations like age of the culture or due to external factors like different medium charges used. As the light control in the main experiment was contaminated after week 2, the anthocyanin concentrations from week 2 were used respectively the ratios between the conditions from week 2 were compared to evaluate the prediction. The ratios from the prediction of the optimized conditions and the actual ratios of the control experiment are shown in Table 6. With the optimized lighting conditions the prediction for week 2 was to reach concentrations that are 2.1-fold (Pn-Glu) respectively 1.9-fold (Cy-Glu) higher than the concentration in the light control or 0.6-fold (Dp-Glu) respectively 0.7-fold (Pt-Glu) lower than the concentration in the light control.

**Table 6:** Predicted and actual ratios between the anthocyanin concentrations of cells cultivated under different lighting conditions.

Goal	Predicted ratio (optimized condition/light control) week 2	Actual ratio (optimized condition/light control) week 2	Goal achieved?
Pn-Glu up	2.1	1.8	Yes, but prediction not reached
Cy-Glu up	1.6	2.4	Yes, better than prediction
Dp-Glu down	0.6	0.5	Yes, better than prediction
Pt-Glu down	0.7	0.3	Yes, better than prediction

Predicted ratios were calculated the following before conducting the control experiment: the anthocyanin concentration in week 2 in cells cultivated under the optimized lighting conditions were predicted using the numerical optimization tool of the Design-Expert software and then divided by the actual anthocyanin concentration in cells cultivated under white light in week 2 of the main experiment. Actual ratios were calculated after analyzing the control experiment: the actual anthocyanin concentration in cells cultivated under optimized lighting conditions in week 2 of the control experiment was divided by the actual anthocyanin concentration in cells cultivated under white light in week 2 of the control experiment.

Conc. = Concentration, Cy = Cyanidin, Dp = Delphinidin, Pn = Peonidin, Pt = Petunidin, Glu = Glucoside, Up = Upregulation (the goal is to maximize the concentration), Down = Downregulation (the goal is to minimize the concentration).

Even though we could not reach the exact values of the prediction, the control experiment showed that with our predicted lighting conditions it is possible to increase (Pn-Glu and Cy-Glu) or decrease (Dp-Glu and Pt-Glu) the anthocyanin concentrations. For Pn-Glu the actual ratio was slightly lower than predicted, but we still achieved 80% more Pn-Glu in week 2 of the cultivation only by applying optimized lighting conditions instead of standard white light, which proofs that LiS can be successfully used to perform DOE-based identification of lighting conditions that lead to optimal yields of selected PSMs. .

This proof was also supported by the Cy-Glu concentrations in cultures that were cultivated under different lighting conditions. The optimized lighting conditions lead to a 2.4-fold higher Cy-Glu concentration in week 2 compared to the light control, which is even better than predicted. With one DoE experiment and one control experiment it is not yet proven that those optimized conditions are the perfect conditions for Pn-Glu and Cy-Glu, because other wavelengths, photoperiods or light intensities may also effect the accumulation, but nevertheless those experiments show the power of LiS and DoE, as we already reached 80% respectively 140% higher anthocyanin concentrations after 2 weeks of cultivation.

Deviations from the predictions can be caused by biological variations of the cultures, because the source cultures from the main and the control experiments were not the same. The source culture used in the control experiment was generated from callus more than a year later than the culture used in the initial experiment. Therefore the callus had been subcultured more often, which could lead to different behavior of the cultures due to genetic cell instability of plant cell cultures.

For Dp-Glu and Pt-Glu downregulation we also reached ratios that are better than predicted, as we achieved 50% less Dp-Glu and even 70% less Pt-Glu in the cells cultivated under optimized conditions. Dp-Glu and Pt-Glu concentrations with no significant differences to the dark controls were measured in the first week and for Pt-Glu also in the second week of the control experiment and in the other weeks the concentrations were only slightly higher than the dark controls, confirming that also a downregulation of anthocyanins is possible by applying the right lighting conditions.

When looking at the ratios between the cultivation conditions in the other weeks of the control experiments and the absolute values of the anthocyanin concentrations (Fig. 5 and Table 6) there are also variations from week to week, which could be caused by flask-to-flask variations due to the weekly subculturing, different aggregation patterns of the cells or overall genetic cell instability^4^. But nevertheless, the target (up- or downregulation) was reached every week with significant differences between the optimized conditions and the light control.

The control experiment confirmed our predictions for four anthocyanins and showed that it is possible to reach higher respectively lower anthocyanin concentrations when using optimized lighting conditions. This was possible even when the source cultures from which the main and the control experiments were started were different.

Further possible control experiments could not only confirm the effect of the optimized lighting conditions, but also point towards differences between the anthocyanins or on differences between the aglycones and the conjugated forms. The data shows a trend that aglycones need more red light than the conjugated forms when the target is to maximize their concentrations, but more optimized lighting conditions need to be checked to strengthen this hypothesis.

The reasons for the effects of different wavelengths on anthocyanin levels are unclear, but the LiS system allows the fast and reliable screening of optimized lighting conditions in PCSCs and could be used to study this phenomenon. For example, LiS could be used to determine how the growth and metabolism of PCSCs representing different plant species is affected by different light wavelengths. The applicability of LiS moreover is not limited to do research on the effect of light on anthocyanins, theoretically every secondary metabolite that is affected by light can be studied, for example other flavonoids, but also terpenoids like carotenoids or mono- and sesquiterpenes, as well as tocopherols^26^. It could also be optimized for use with other phototrophic organisms cultivated in Erlenmeyer flasks, such as cyanobacteria or algae. Cyanobacteria are strongly affected by different wavelengths of light^27^.

Our initial proof-of-principle experiment combined with the control experiment have already demonstrated the power and practical application of our LiS system by generating cultures that produce specific anthocyanin concentrations and compositions in response to carefully optimized lighting conditions without adjusting other parameters or applying more advanced strategies such as elicitation or genetic engineering. The LiS system is therefore likely to be even more versatile when combined with these additional approaches. Our study demonstrated that LiS is a highly innovative and useful tool providing for the first time the possibility to screen up to 12 lighting conditions on PCSCs in parallel. LiS allows the individual control of light spectrum, intensity and photoperiod for each Erlenmeyer flask.

## Methods

### Cell line cultivation

*Vitis vinifera* callus cells were obtained from the German Collection of Microorganisms and Cell Cultures (DSMZ, Braunschweig, Germany). Callus cells were subcultured every 4 weeks on Gamborg’s B5 medium^28^ supplemented with 30 g/L sucrose, 0.1 mg/L α-naphthalene acetic acid, 0.2 mg/L kinetin, 0.25 g/L casein hydrolysate and 8 g/L agar (all medium components from Duchefa Biochemie, Haarlem, Netherlands). The pH was adjusted to 5.5 before autoclaving. Callus cells were cultivated at room temperature (20–23 °C) with a 16-h photoperiod (white light, 90 µmol m^−2^ s^−1^). Suspension cultures were established by resuspending a 1-cm fragment of callus in liquid Gamborg’s B5 medium (as above without agar). Routine suspension cultures were subcultured weekly by transferring the cells to fresh medium, and adjusting to 20% of the final volume. The cells were cultivated at 26 °C shaking at 140 rpm with a 16-h photoperiod (white light, 80 µmol m^−2^ s^−1^).

For the experiment, 26 suspension cultures were inoculated from the same routine suspension culture and adjusted to 15% cells (v/v) to ensure the basic conditions were equivalent. Of those 26 cultures, 24 were cultivated in the LiS system under the lighting conditions shown in section 5.4 with a 16-h photoperiod, one (light control) was cultivated under routine conditions (16-h photoperiod, white light, 80 µmol m^−2^ s^−1^) whereas the remaining culture was cultivated in darkness (dark control). All cultures were cultivated at 26 °C and 140 rpm for 4 weeks. Every week, cells were transferred to fresh medium, adjusted to 15% (v/v), and the remaining cells were used for further analysis.

For the control experiment 36 suspension cultures were inoculated from a new routine suspension culture (16-h photoperiod, white light, 80 µmol m^−2^ s^−1^) with 15% cells (v/v) so that the basic conditions of the cultures were equivalent. As the routine suspension culture used in the initial experiment did not exist anymore, we had to use a different routine suspension culture that was prepared from the callus routine in the same way the old routine suspension was, the only difference was the time point of the establishment, meaning that the callus had been subcultured more often. The lighting conditions for the control experiment were calculated by Design-Expert as shown in Table 4. Six replicates were used for each lighting condition, resulting in 24 suspension cultures that were cultivated within the LiS as the empirical proof for our models. We included six replicates as light controls, cultivated under routine conditions (16-h photoperiod, white light, 80 µmol m^−2^ s^−1^) and six replicates as dark controls, cultivated in darkness. All 36 suspension cultures were cultivated at 26 °C and 120 rpm for 4 weeks. Weekly subculturing and harvesting of the cells for analysis was done the same way as in the main experiment.

### Biomass determination

Every week during subcultivation, the PCV of the cultures was determined to measure their growth. PCV is defined as the ratio of the volume of cells and the volume of medium after centrifugation of 10 mL suspension culture (200 g, 5 min). At the beginning of each week of cultivation, every culture had a PCV of 15%.

### Liquid chromatography-ion mobility separation-high resolution mass spectrometry (LC-IMS-HRMS)

The cells that were not used for subculturing were harvested by Miracloth filtration and frozen at −20 °C. LC-IMS-HRMS samples were prepared by disrupting 1 g plant cells mixed with 4 mL 49.5/49.5/1 (v/v/v) water/methanol/formic acid in an ultrasonic bath for 15 min. The mixture was incubated on a horizontal shaking platform (120 rpm, 5 min), followed by centrifugation (2147 g, 5 min). The supernatant was transferred to a separate vial and the extraction step was repeated with the pellet. Both supernatants were combined, shaken by hand and transferred to LC vials for analysis.

The samples were analyzed by ultra-high performance liquid chromatography (UHPLC) followed by ion mobility high resolution mass spectrometry (IMS-HRMS) with a UniSpray ion source in positive ionization mode (USI +), hereinafter LC-IMS-HRMS. Anthocyanin standards with defined concentration were used for a semi-quantitative analysis to generate the anthocyanin profiles of the samples. We analyzed the six most common anthocyanidins (aglycones) in higher plants (Table 7) and five of their glycosides (glucosides, diglucosides, acetylglucosides and coumaroylglucosides) each.

**Table 7:** The six most common anthocyanidins in higher plants^18,19^.

Name	Proportion [%] in higher plants	Color	Occurrence
Cyanidin (Cy)	50	Orange-red–purple (magenta)	Major pigment in berries and red-colored vegetables
Delphinidin (Dp)	12	Bluish-red	Blue hue in flowers
Pelargonidin (Pg)	12	Orange	Orange hue in flowers, red hue in some fruits and berries
Peonidin (Pn)	12	Orange-red–purple (magenta)	In berries, grapes and red wines
Malvidin (Mv)	7	Bluish-red	In blue-colored flowers, major red pigment in red wines
Petunidin (Pt)	7	Bluish-red	In blackcurrants and purple petals of flower

### Design of experiments

We planned the different lighting conditions using a DoE (IV-optimal design RSM) in Design-Expert (Design-Expert® software, version 11.0.3, Stat-Ease, Inc., Minneapolis, MN, USA, www.statease.com). We used a quadratic model design with 12 model points, 5 lack-of-fit points, 5 replicate points and an additional center point resulting in 24 runs (= 24 individual LiS vessels) as shown in Table 1.

The intensity of red, green and blue light was analyzed as discrete numeric factors and planned as a mixture design, so that the sum of those factors was constant. UV light was analyzed as a nominal categoric factor (on/off). After reviewing the anthocyanin LC-IMS-HRMS data we decided to expand the model by including the cultivation time “week” as a discrete factor with four levels. The levels represent the time points of subculturing and harvesting the cells for anthocyanin analysis. Because this factor was included after the experiment was complete, we used the historical data function in Design-Expert to analyze the results. The mixture components, factor ranges, types and levels are shown in Table 2.

After including the cultivation time and switching to historical data, the model resulted in 96 runs, consisting of the original 24 runs in each of the 4 weeks.

Statistical relationships were determined using Design-Expert v11.0.3. ANOVA was carried out and the correlation coefficient R^2^ was determined for all anthocyanins. We also used Design-Expert’s numerical optimization function, which allows certain goals and restrictions to be set for every factor and response. Therefore we set the goals to find the lighting conditions that minimize and maximize the concentration of every anthocyanin tested, as well as various groups of anthocyanins. The only restrictions for factors A-E were to be within the range of the original design. Four predicted lighting conditions were tested in a control experiment. An ANOVA was applied to the PCV data to find a correlation between the lighting conditions and the growth of the cultures using GraphPad Prism v8.4.3 (GraphPad Software, San Diego, CA, USA).

## Supplementary Information

Supplementary Information.

## Data Availability

The datasets used and/or analyzed during the current study are available from the corresponding author on reasonable request.
