# Supplementary material for: LEDitSHAKE: a lighting system to optimize the secondary metabolite content of plant cell suspension cultures
Source: Sci Rep. 2021 Dec 2;11:23353. doi: 10.1038/s41598-021-02762-6 (PMC8639678; doi:10.1038/s41598-021-02762-6)
Supplement: Supplementary file 1 — Supplementary Information. [file 41598_2021_2762_MOESM1_ESM.pdf]

LEDitSHAKE – A Lighting System to Optimize the Secondary Metabolite Content of Plant Cell Suspension Cultures

Ann-Katrin Beuel*, Natalia Jablonka, Julia Heesel, Kevin Severin, Holger Spiegel, Stefan Rasche

Ann-Katrin Beuel (corresponding author; ann-katrin.beuel@ime.fraunhofer.de), Natalia Jablonka, Julia Heesel, Holger Spiegel and Stefan Rasche:

Fraunhofer Institute for Molecular Biology and Applied Ecology IME,

Forckenbeckstraße 6, Aachen, 52074, Germany

Kevin Severin:

Fraunhofer Institute for Molecular Biology and Applied Ecology IME,

Auf dem Aberg 1, Schmallenberg, 57392, Germany

Supplementary Information

Supplementary Figures 1 – 36: Concentrations of analyzed anthocyanins in grapevine cultures under different lighting conditions at different time points. See the top of each slide for the anthocyanin to which the data refer. All cultures originated from the same source culture. All 26 replicate cultures were cultivated for 4 weeks at 26 °C, shaking at 140 rpm. Every week, 15% of the cells (v/v) were subcultured and the remaining cells were collected for LC-IMS-HRMS analysis to determine anthocyanin concentrations. Each line represents a lighting condition: conditions 1–24 are based on the DoE model (Table 1) with an intensity of red + green + blue light = 50 µmol m^{-2} s^{-1} and UV light either off or on for 1 h (12–1 pm) each day (9 µmol m^{-2} s^{-1}). Those cultures were cultivated in the LiS under 24 different lighting conditions with a 16-h photoperiod. The light control was cultivated according to the conditions of the routine/source culture (standard shaking incubator, light positioned in the ceiling, 16-h photoperiod, 80 µmol m^{-2} s^{-1}), but was contaminated after 3 weeks, therefore the data represent only 2 weeks of cultivation. The dark control was cultivated in the absence of light.

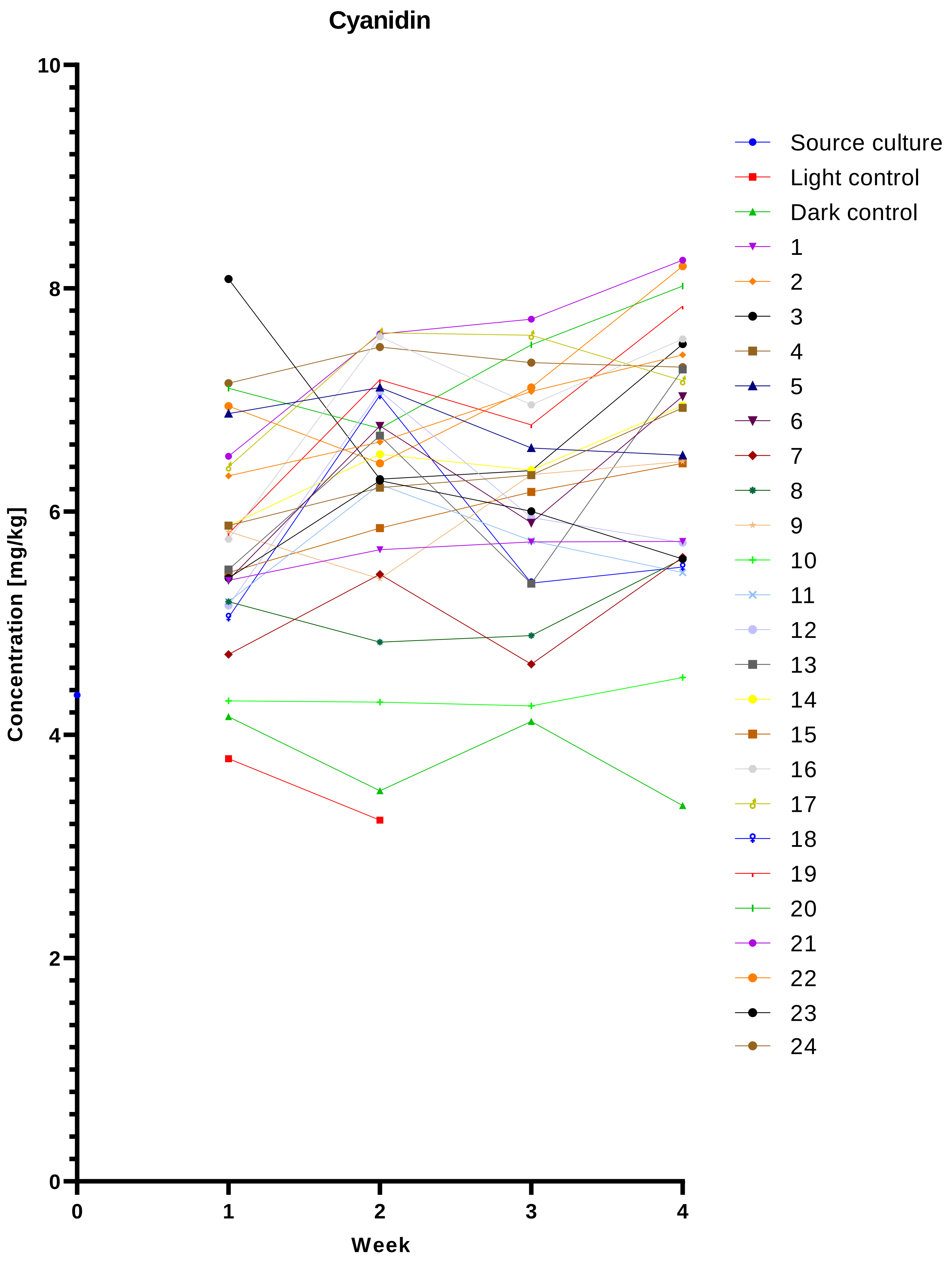

Supplementary Figure 1

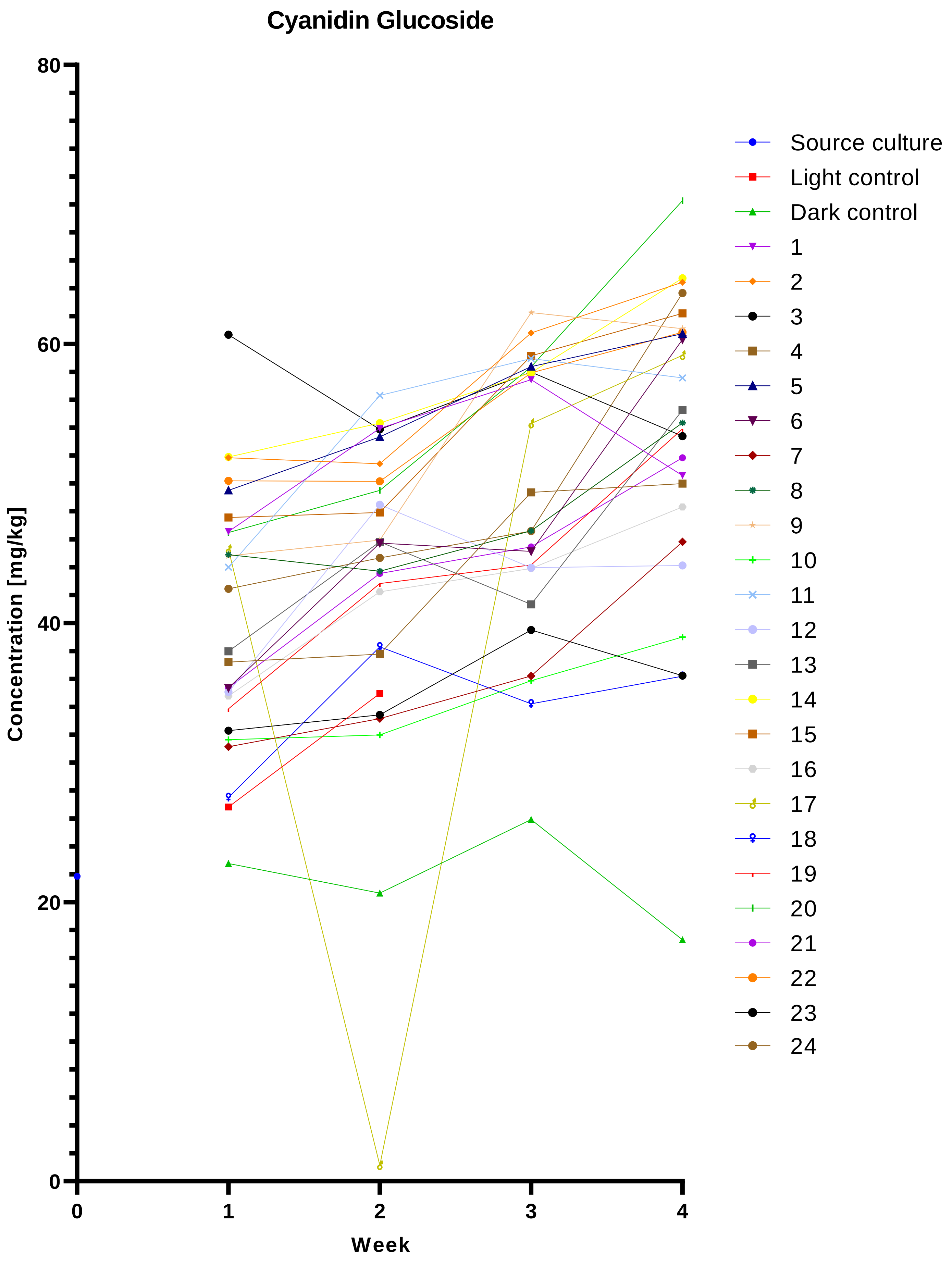

Supplementary Figure 2

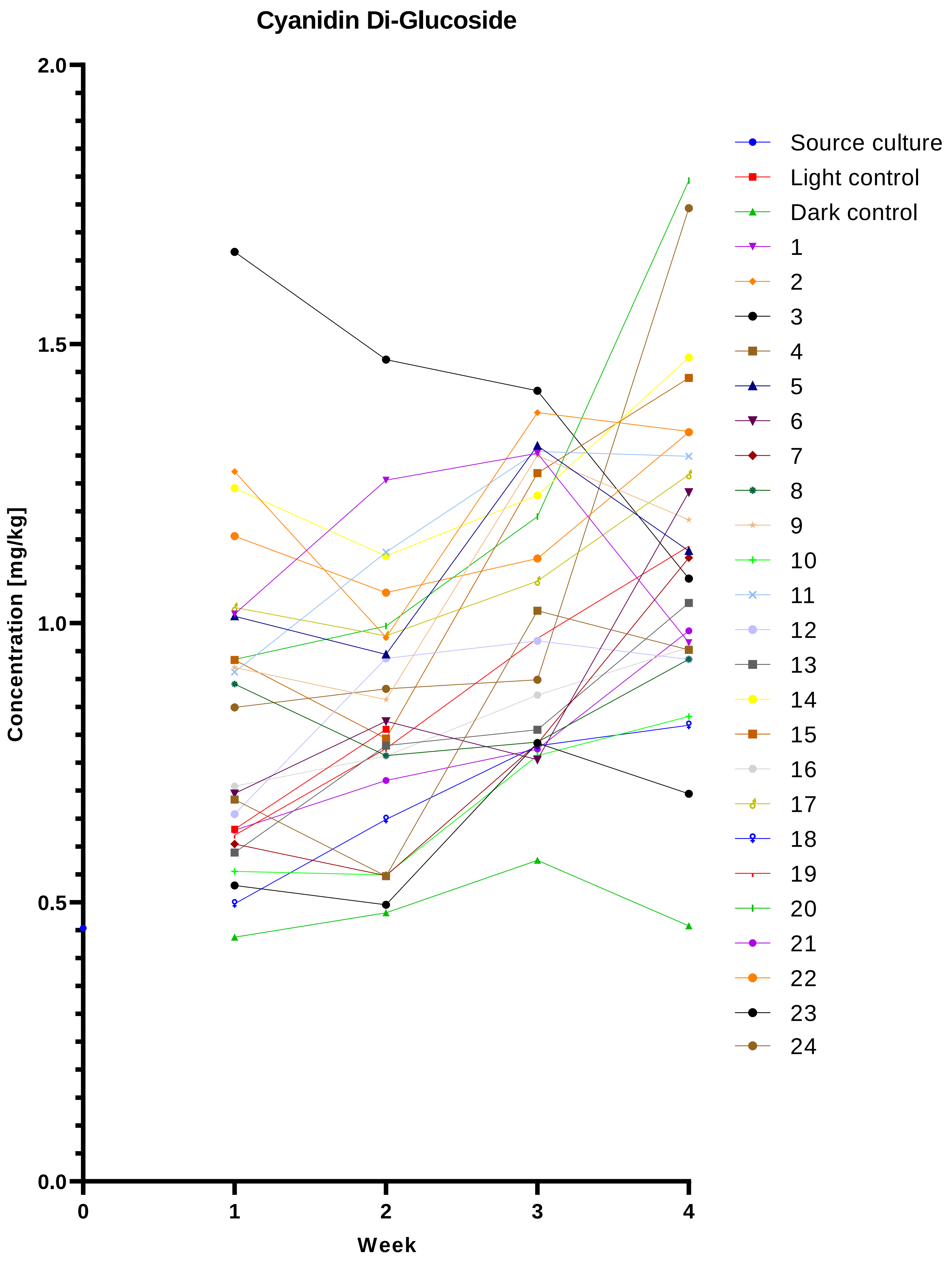

Supplementary Figure 3

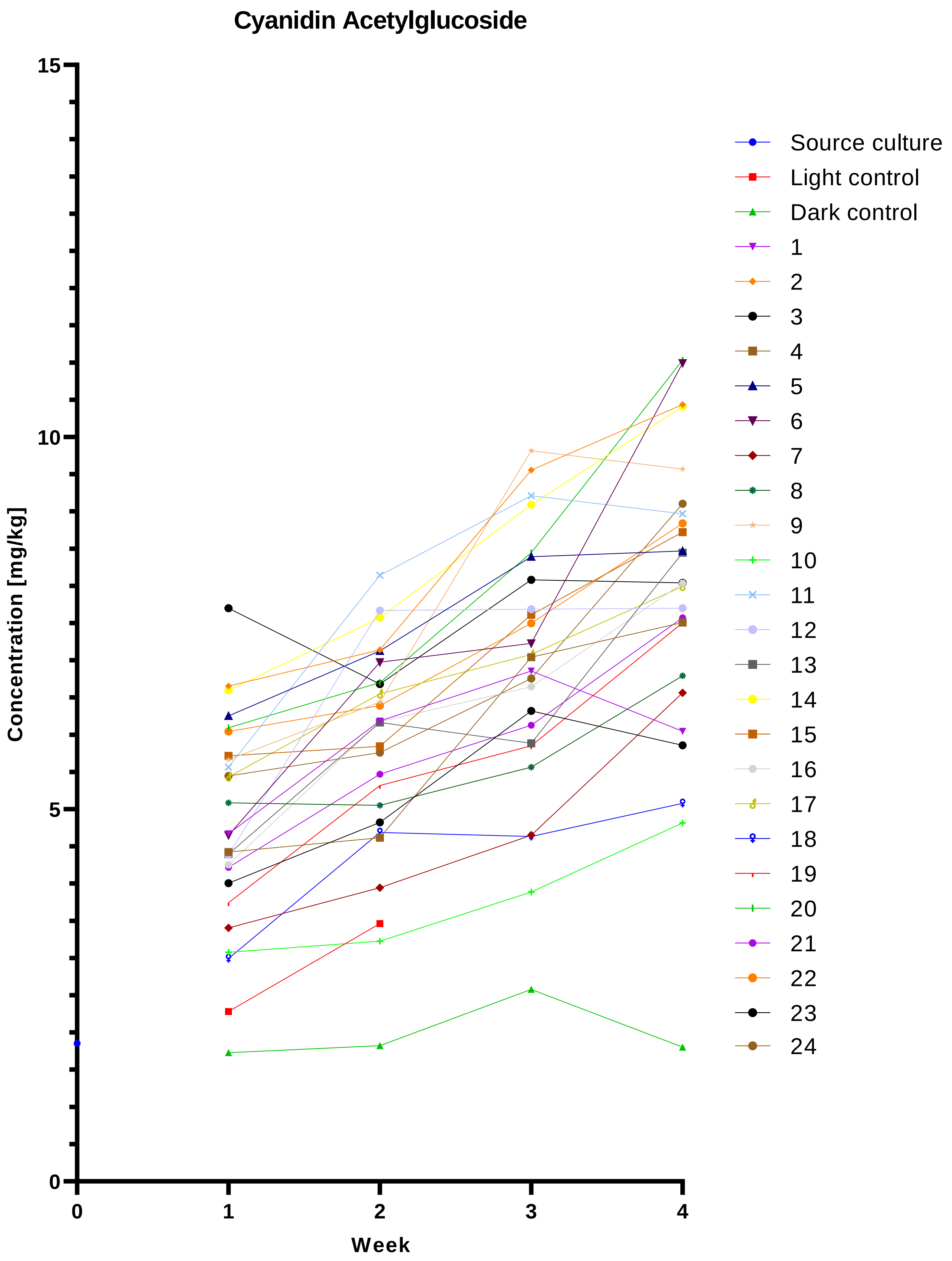

Supplementary Figure 4

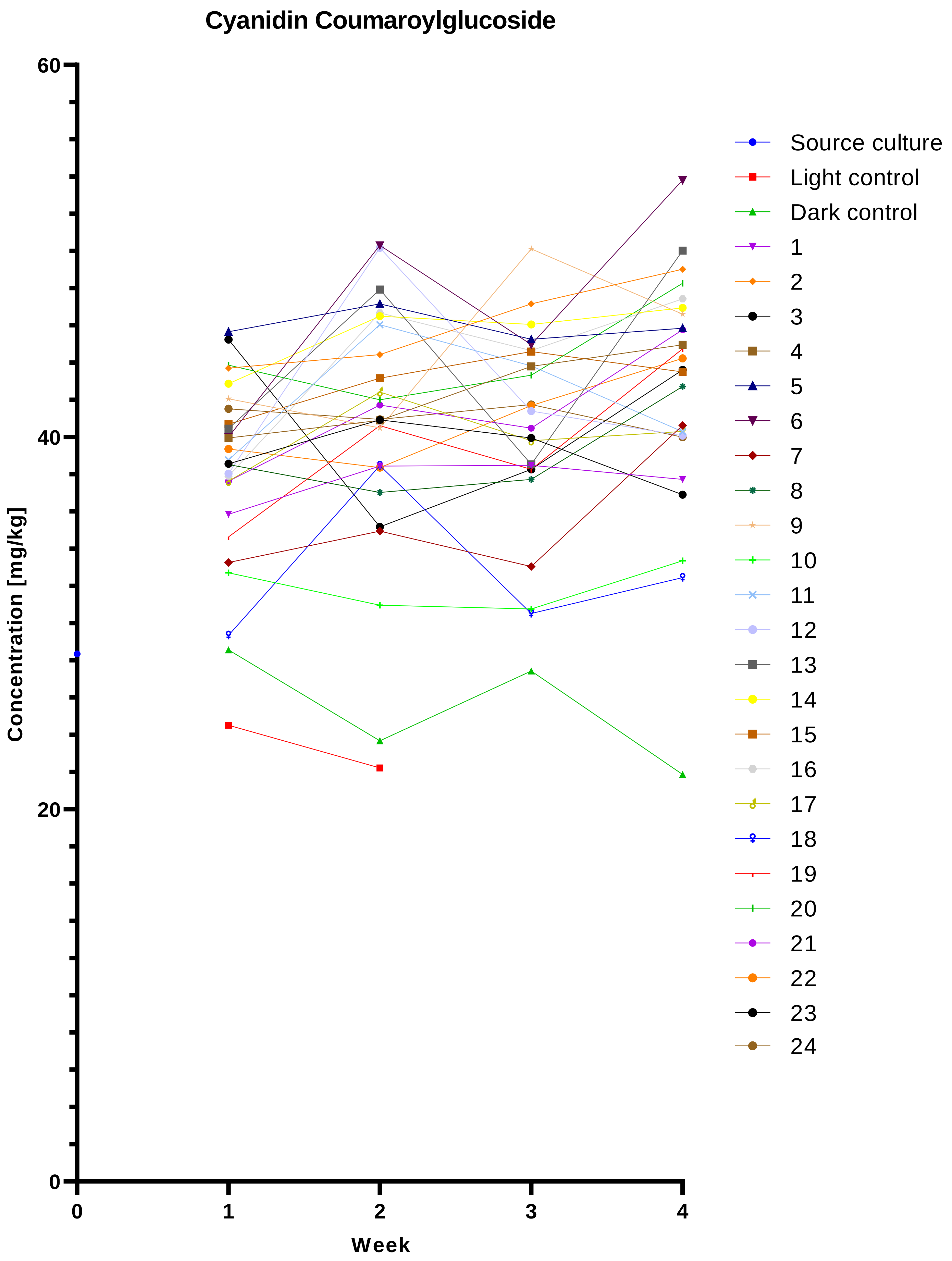

Supplementary Figure 5

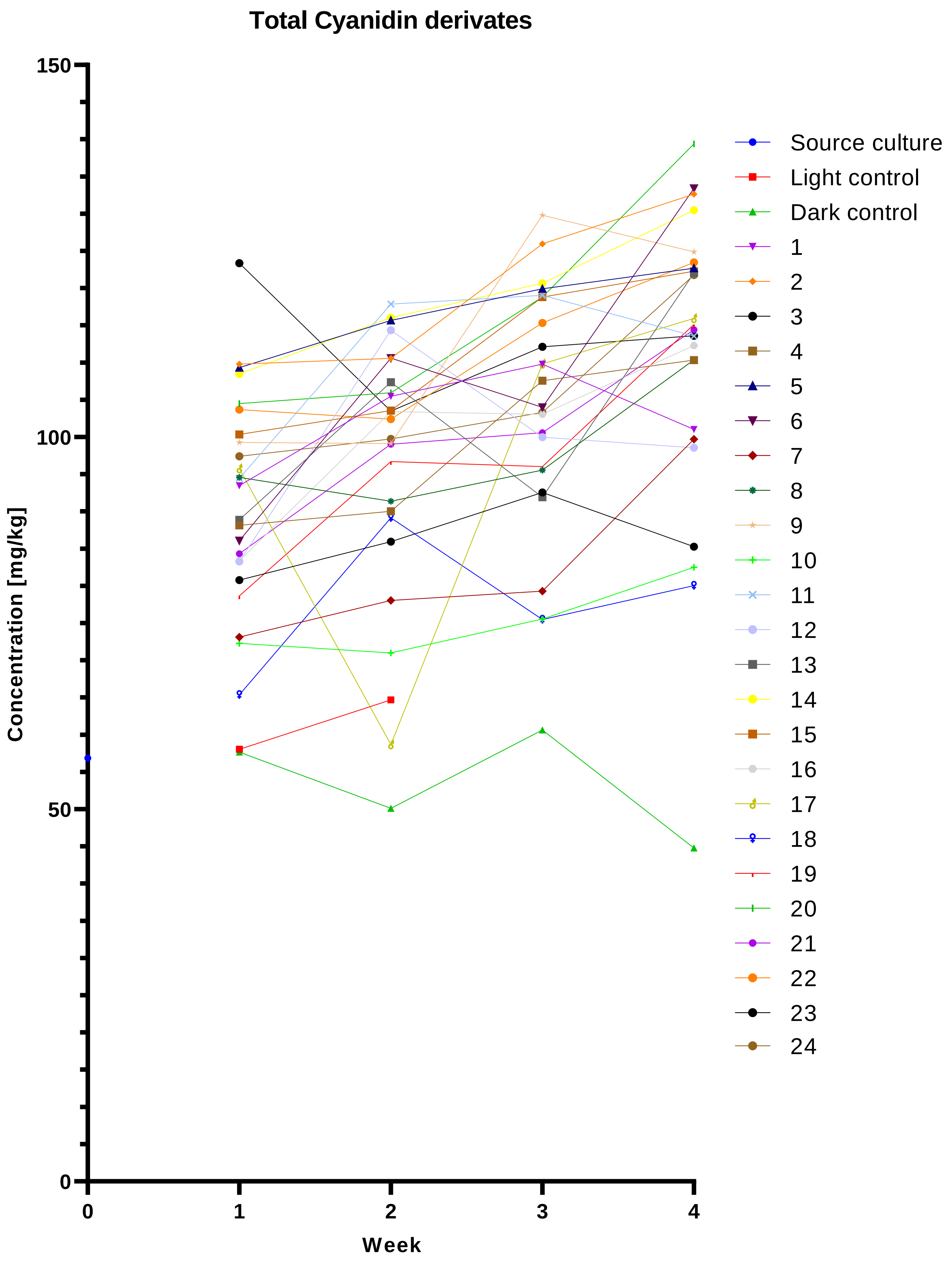

Supplementary Figure 6

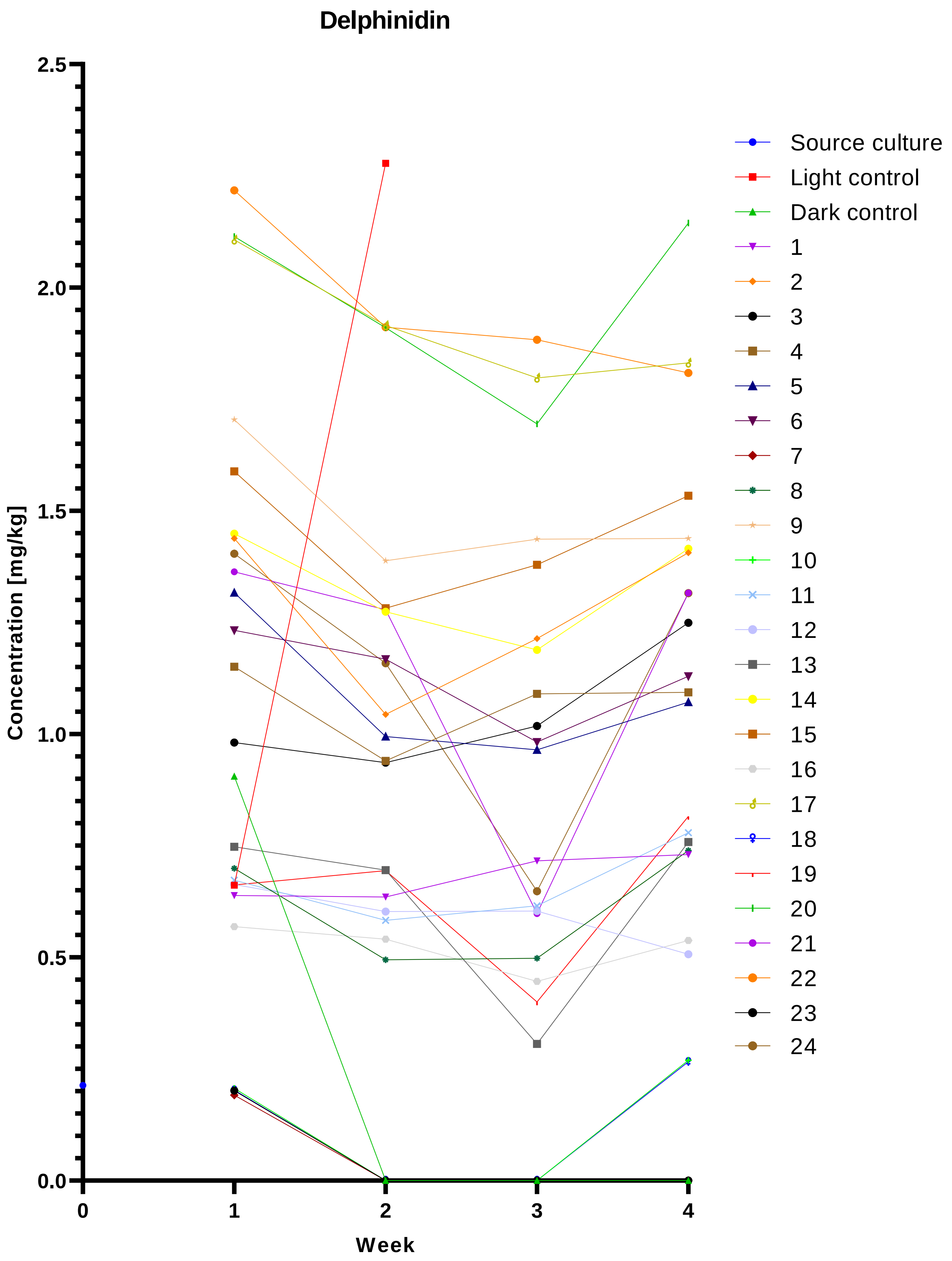

Supplementary Figure 7

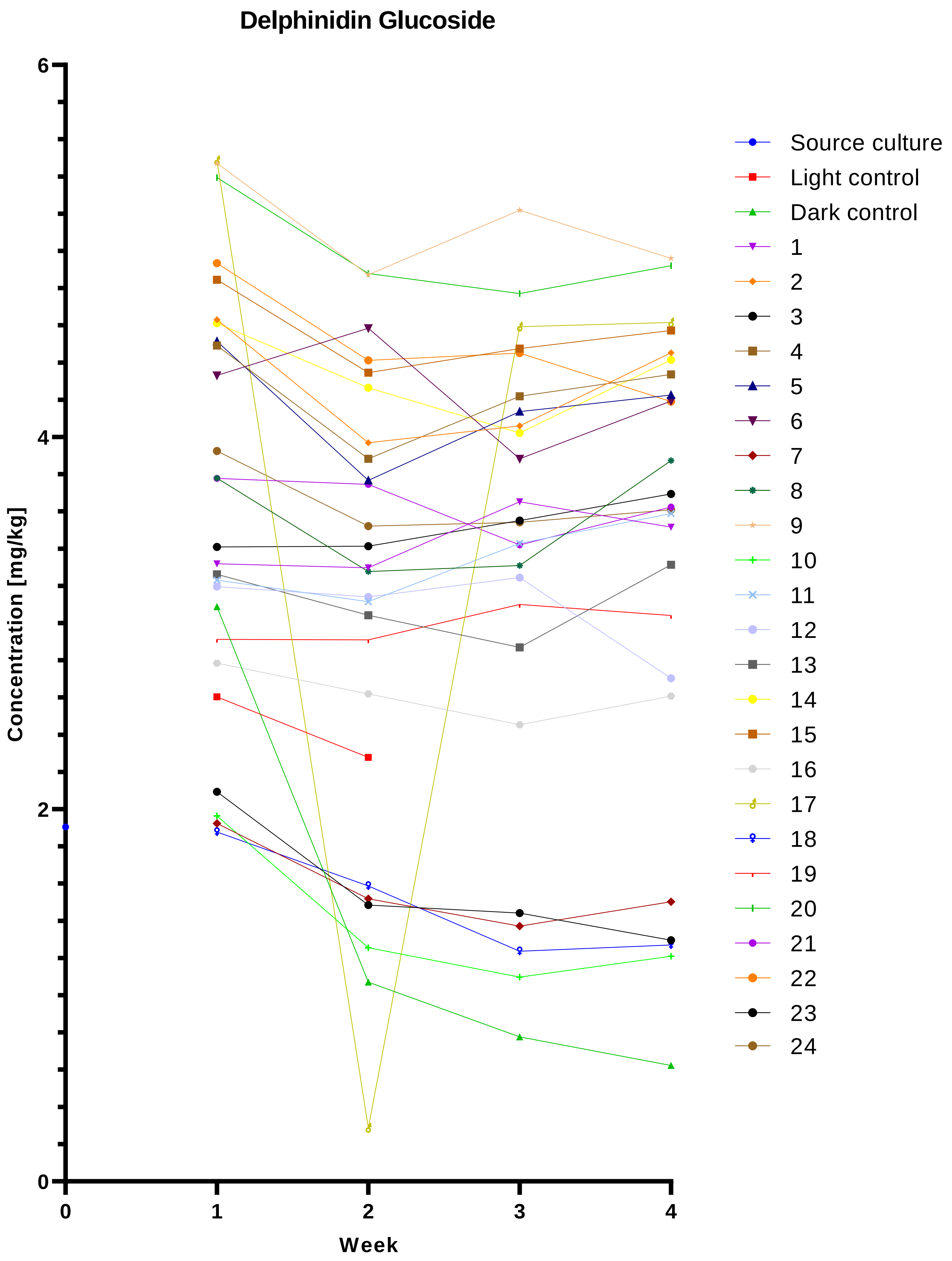

Supplementary Figure 8

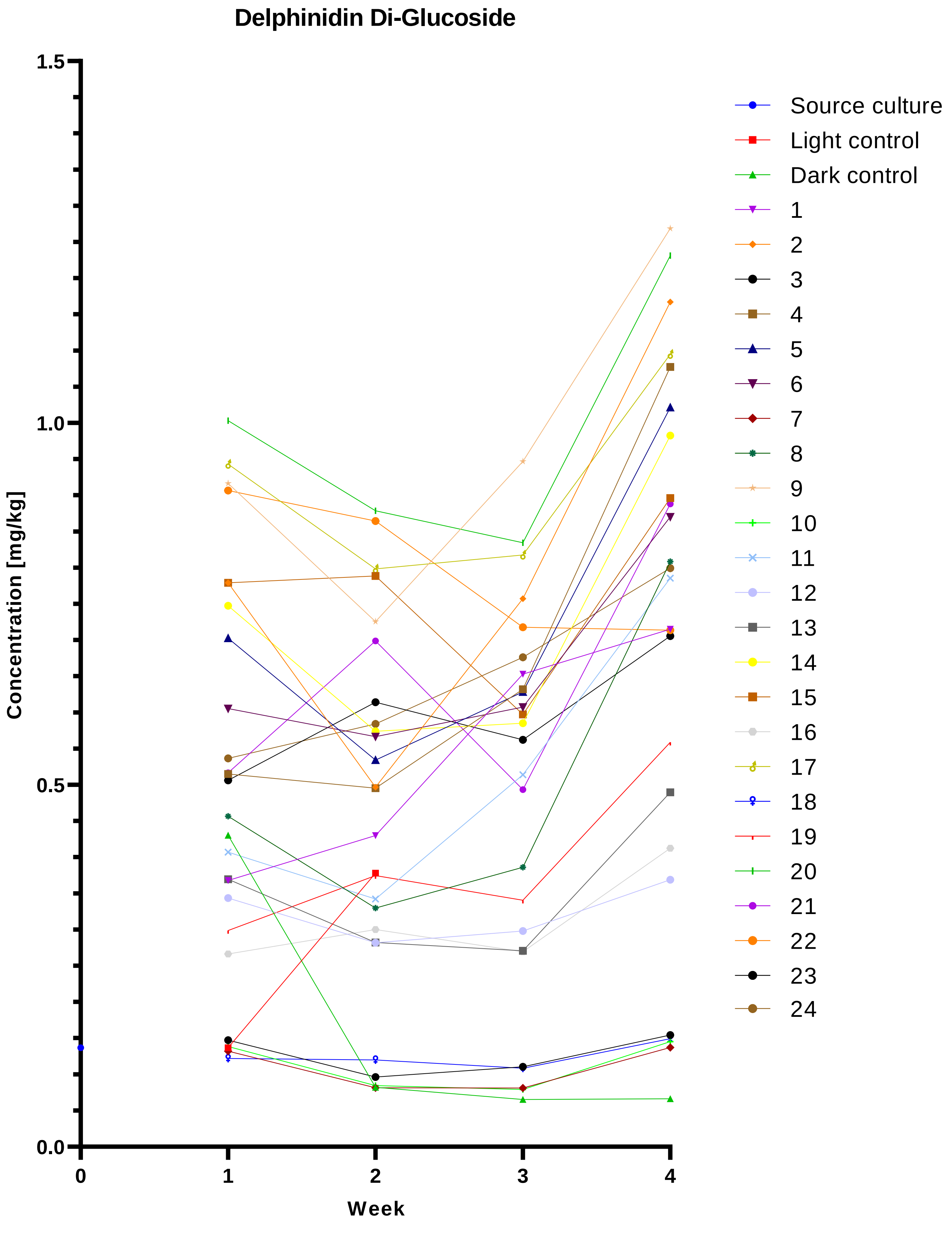

Supplementary Figure 9

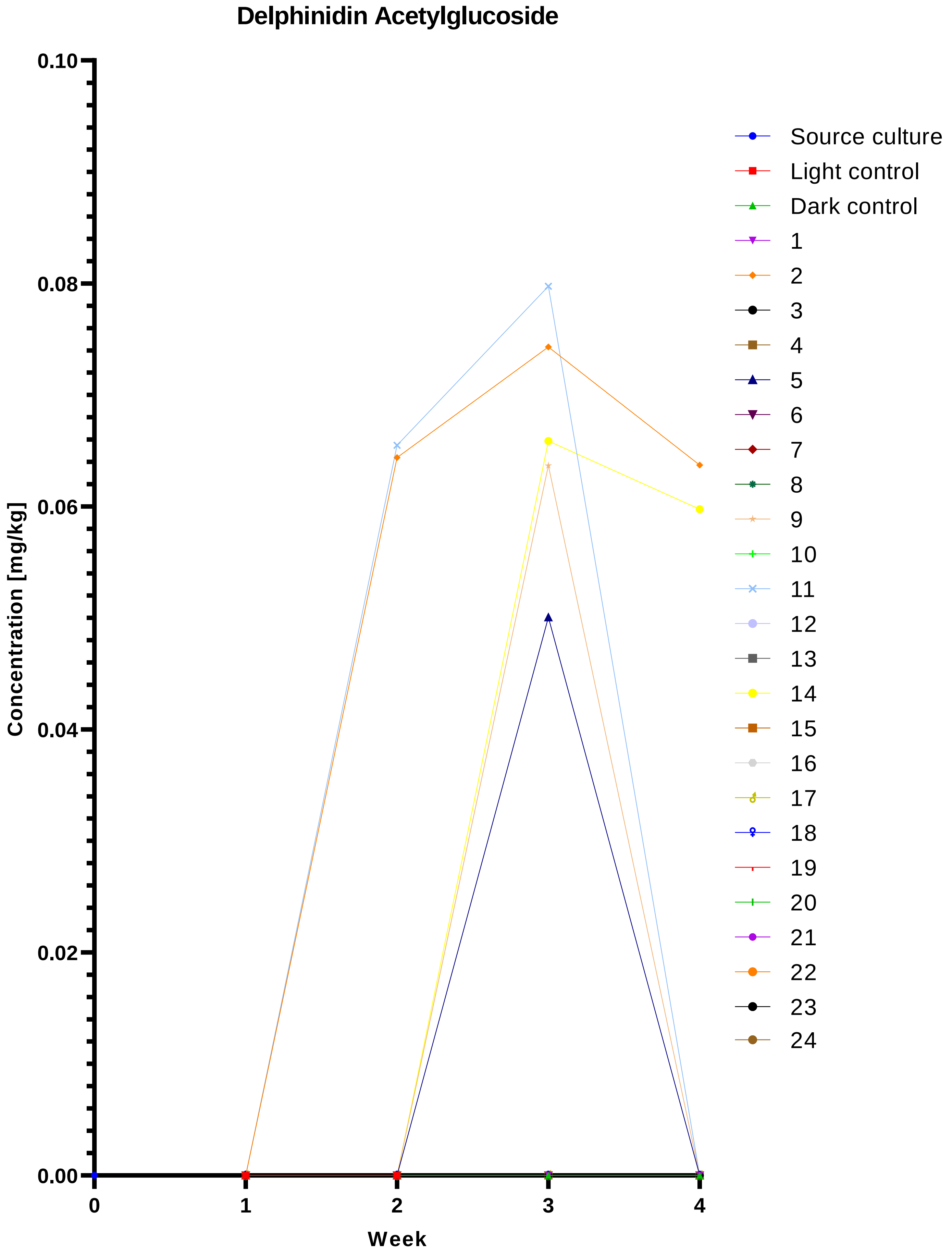

Supplementary Figure 10

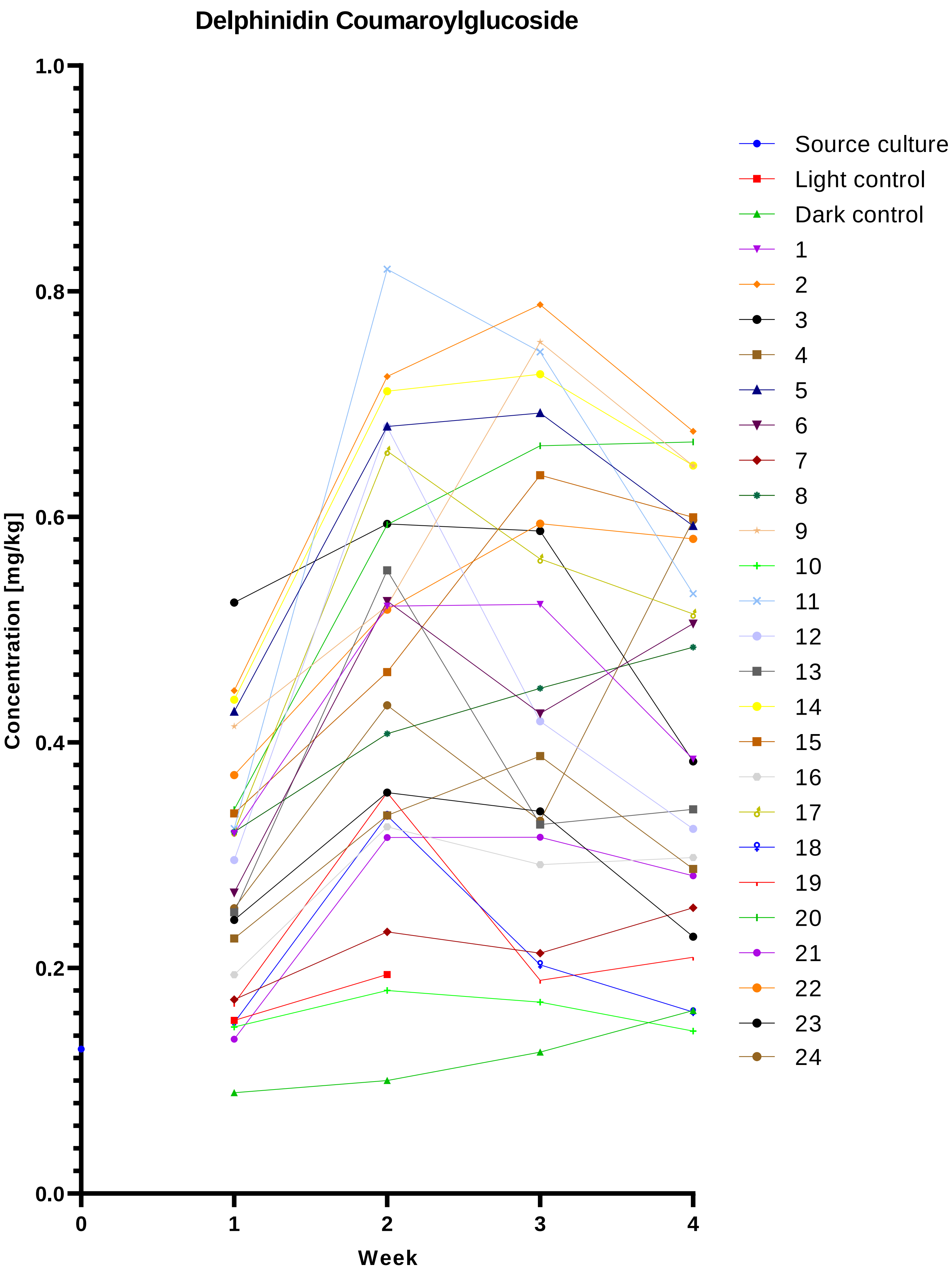

Supplementary Figure 11

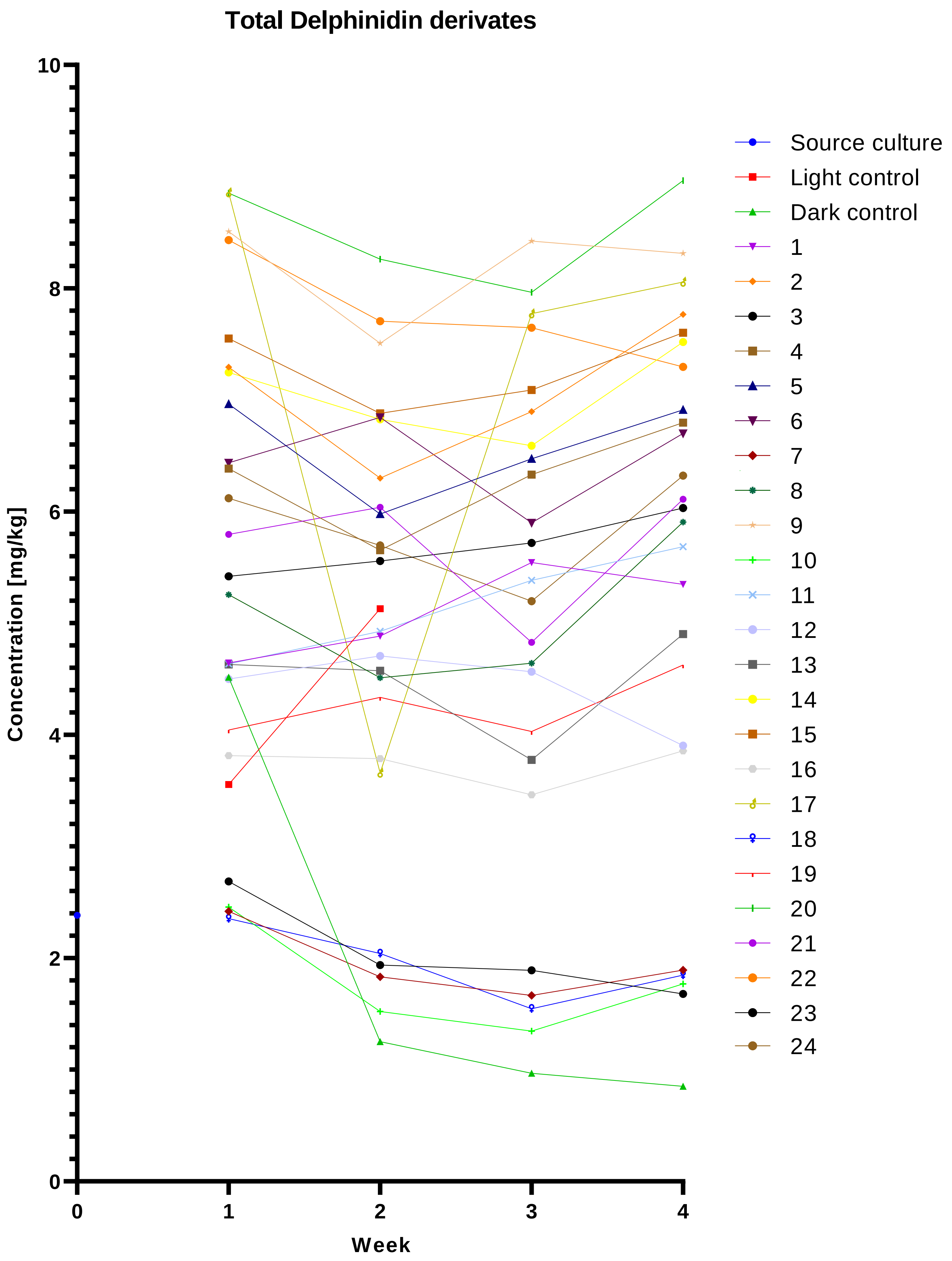

Supplementary Figure 12

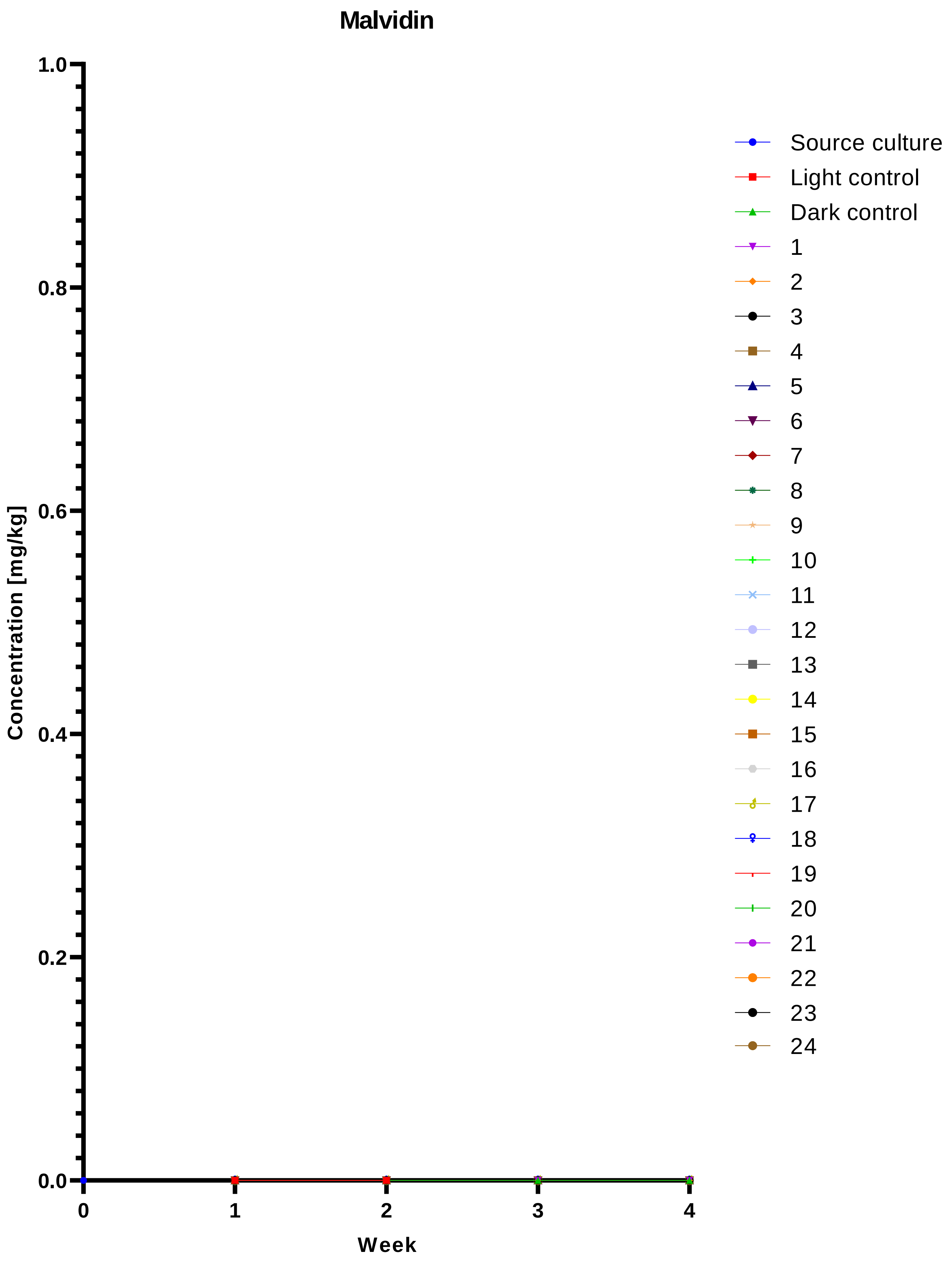

Supplementary Figure 13

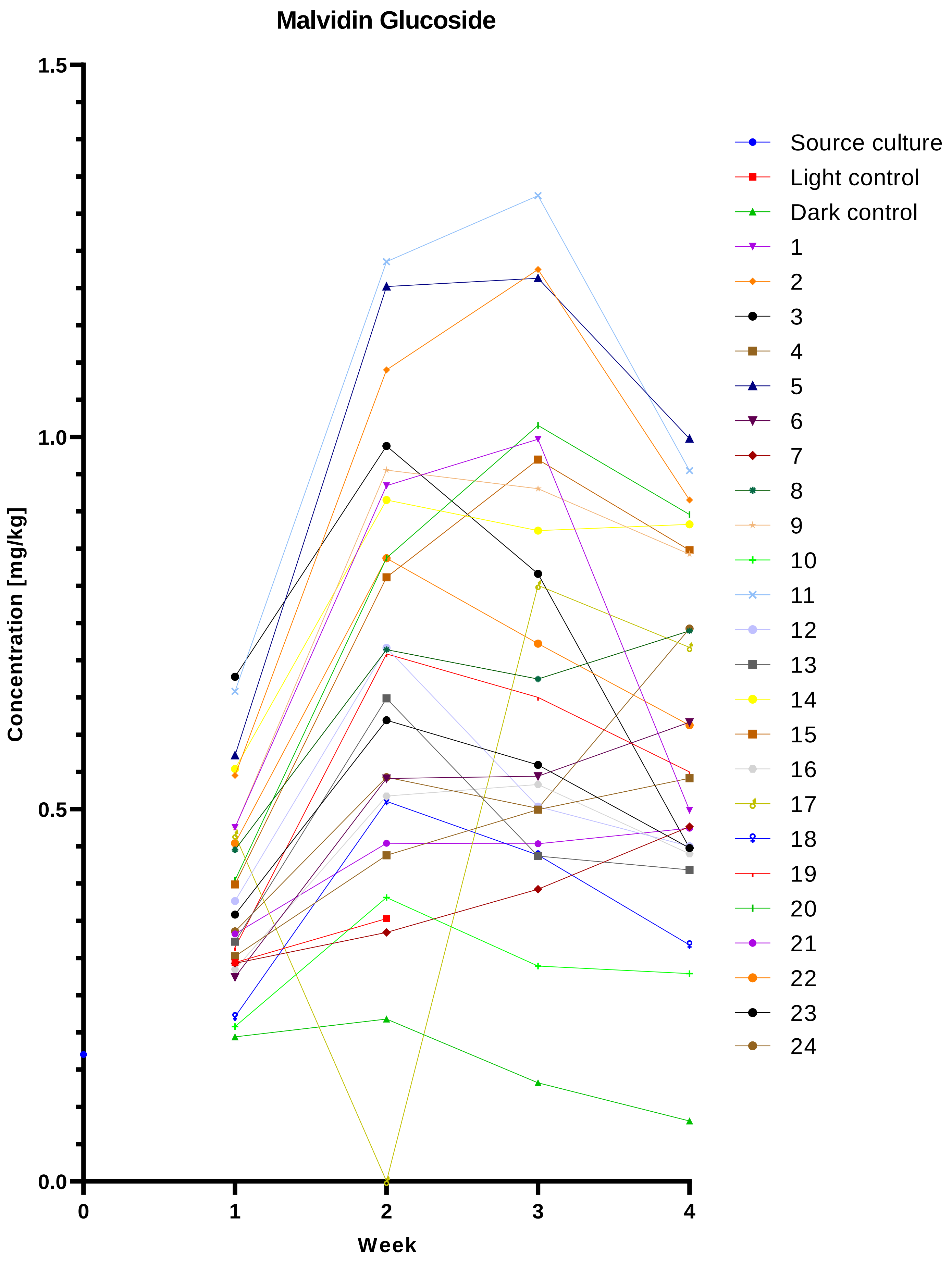

Supplementary Figure 14

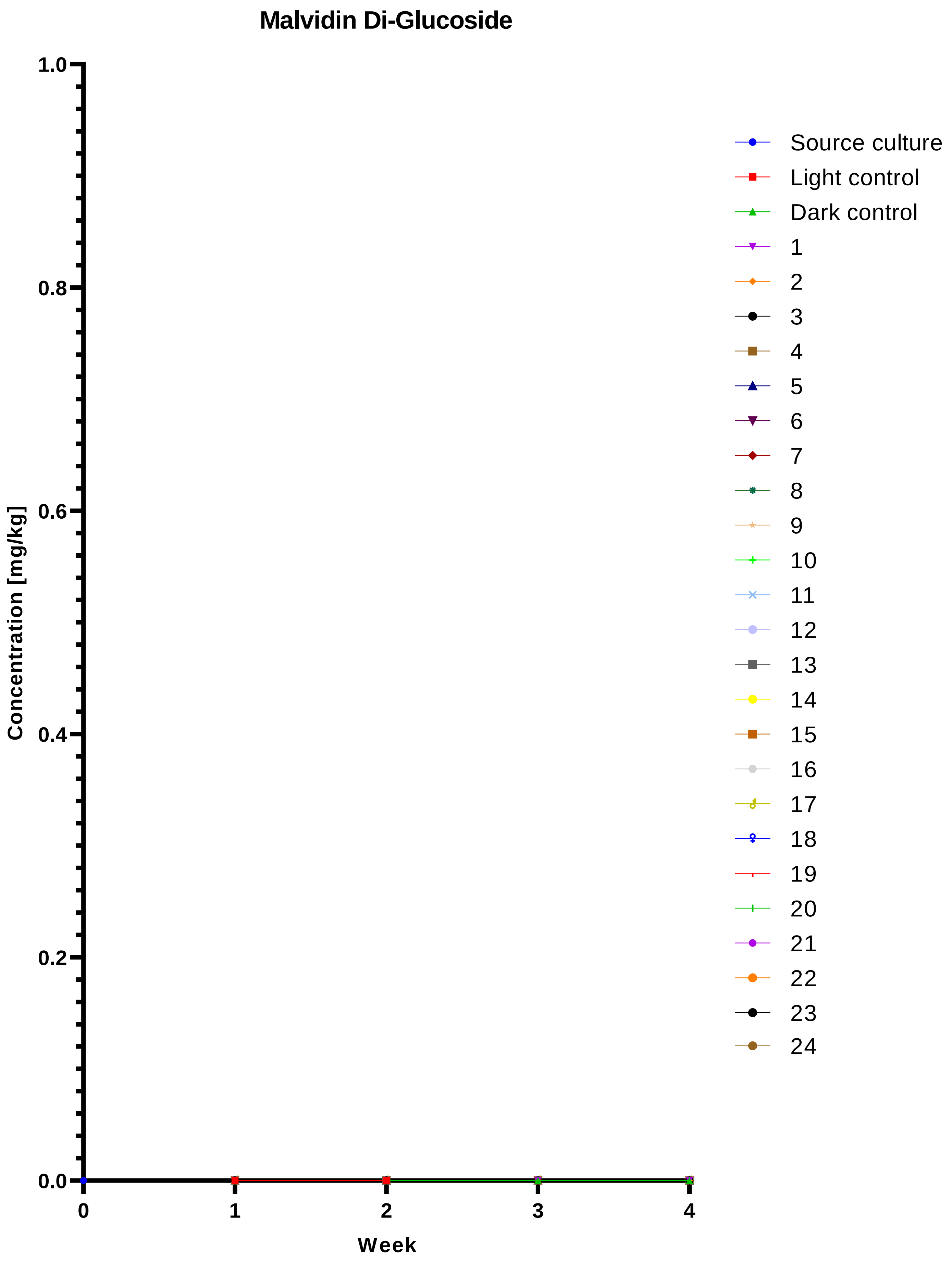

Supplementary Figure 15

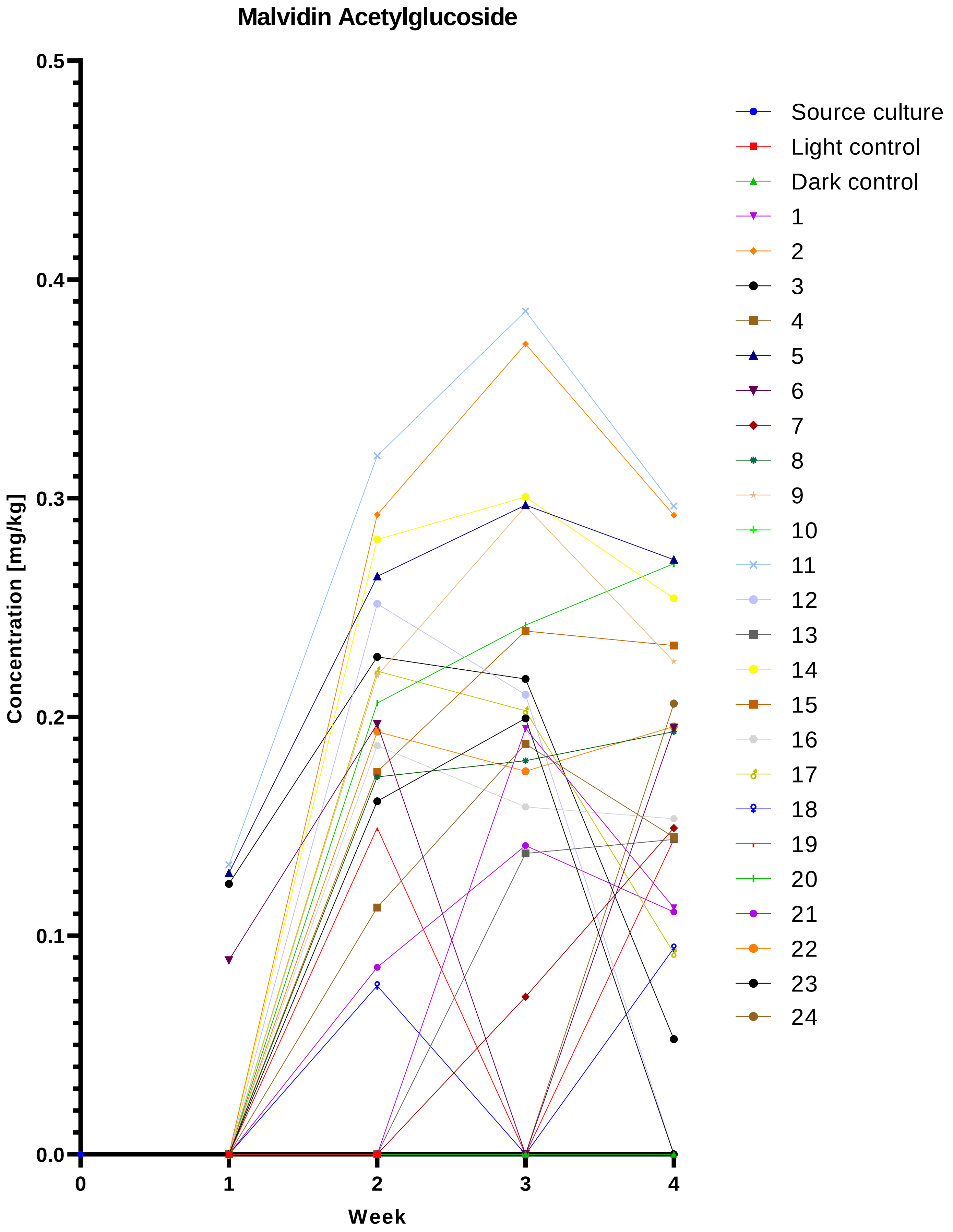

Supplementary Figure 16

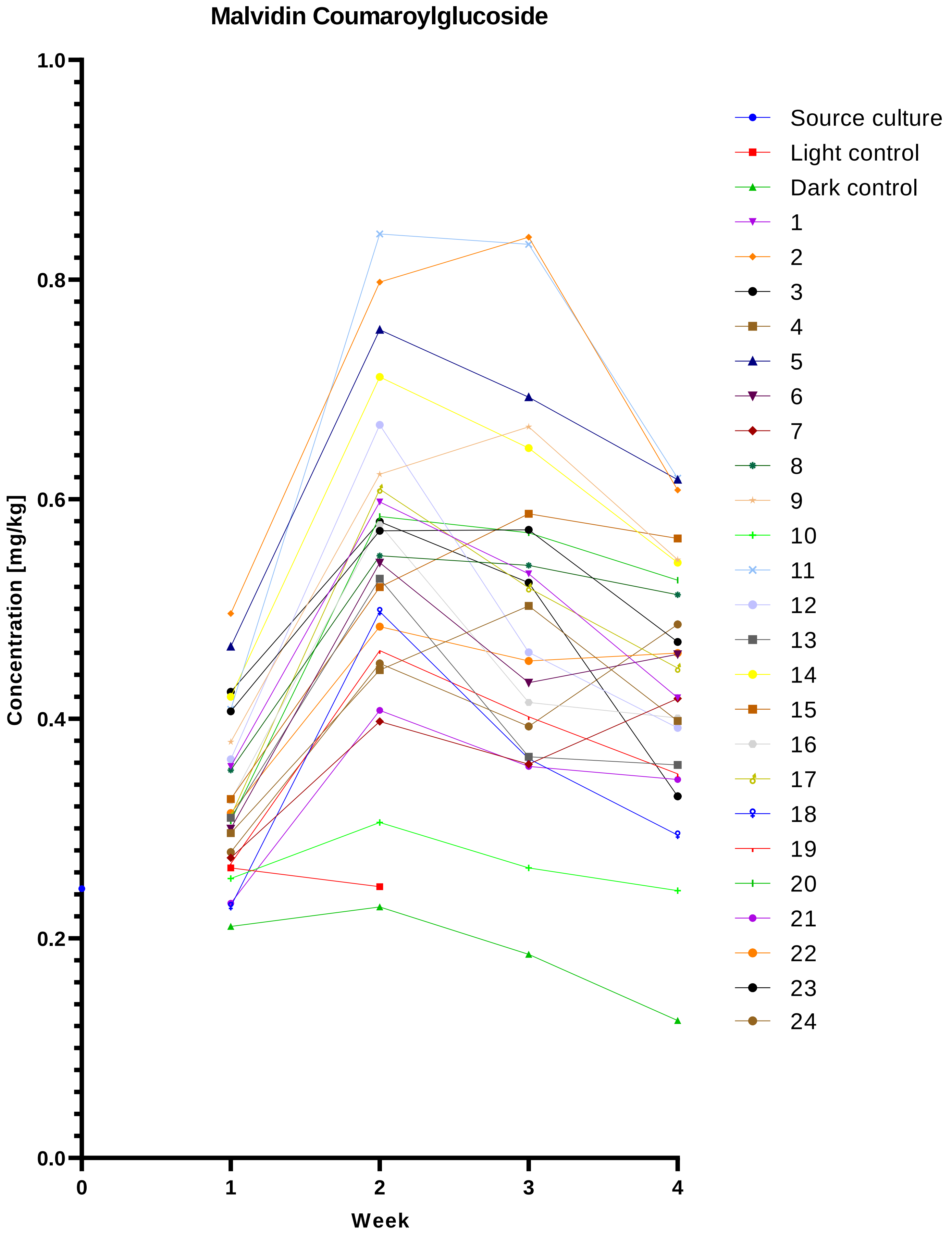

Supplementary Figure 17

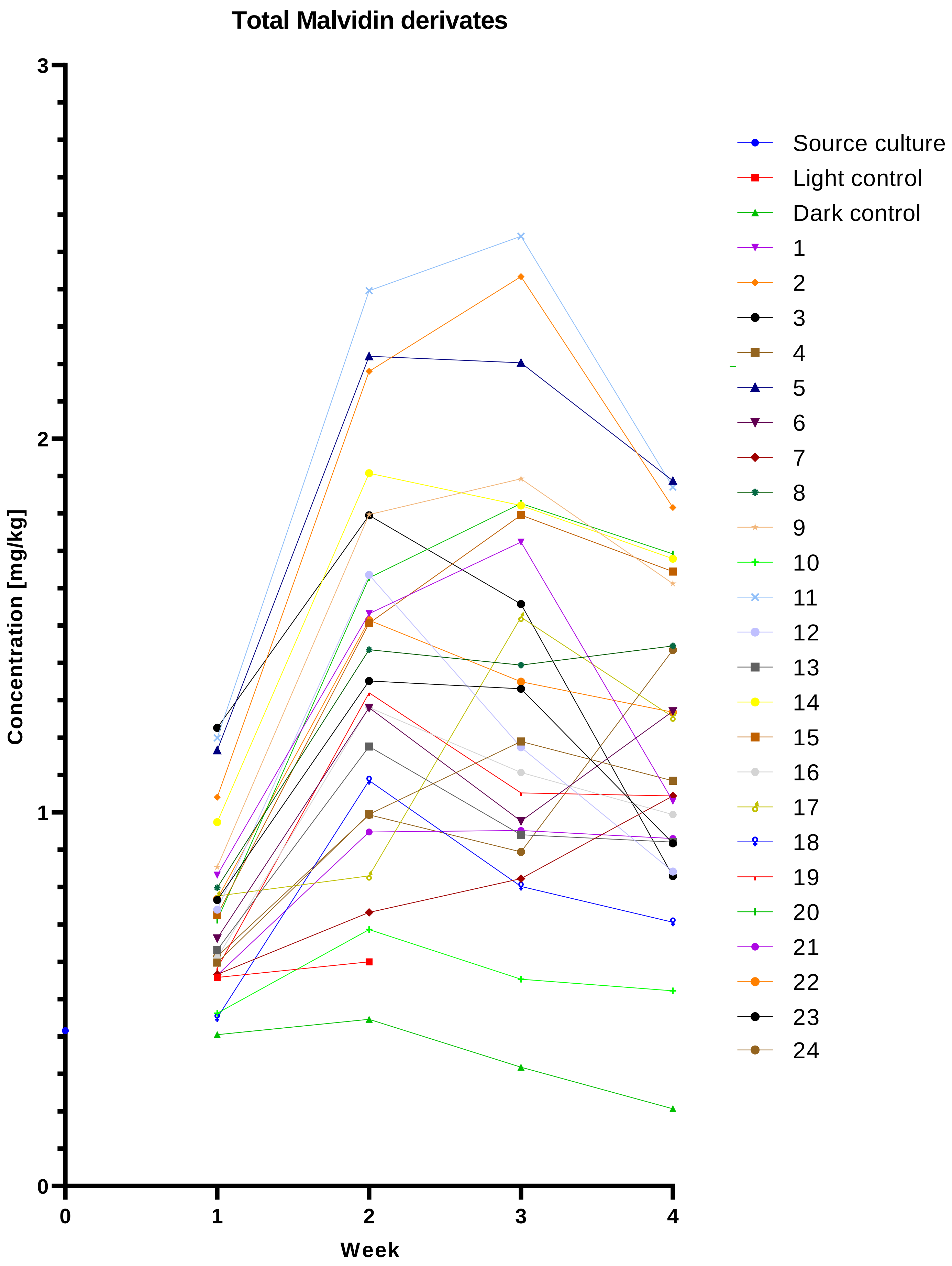

Supplementary Figure 18

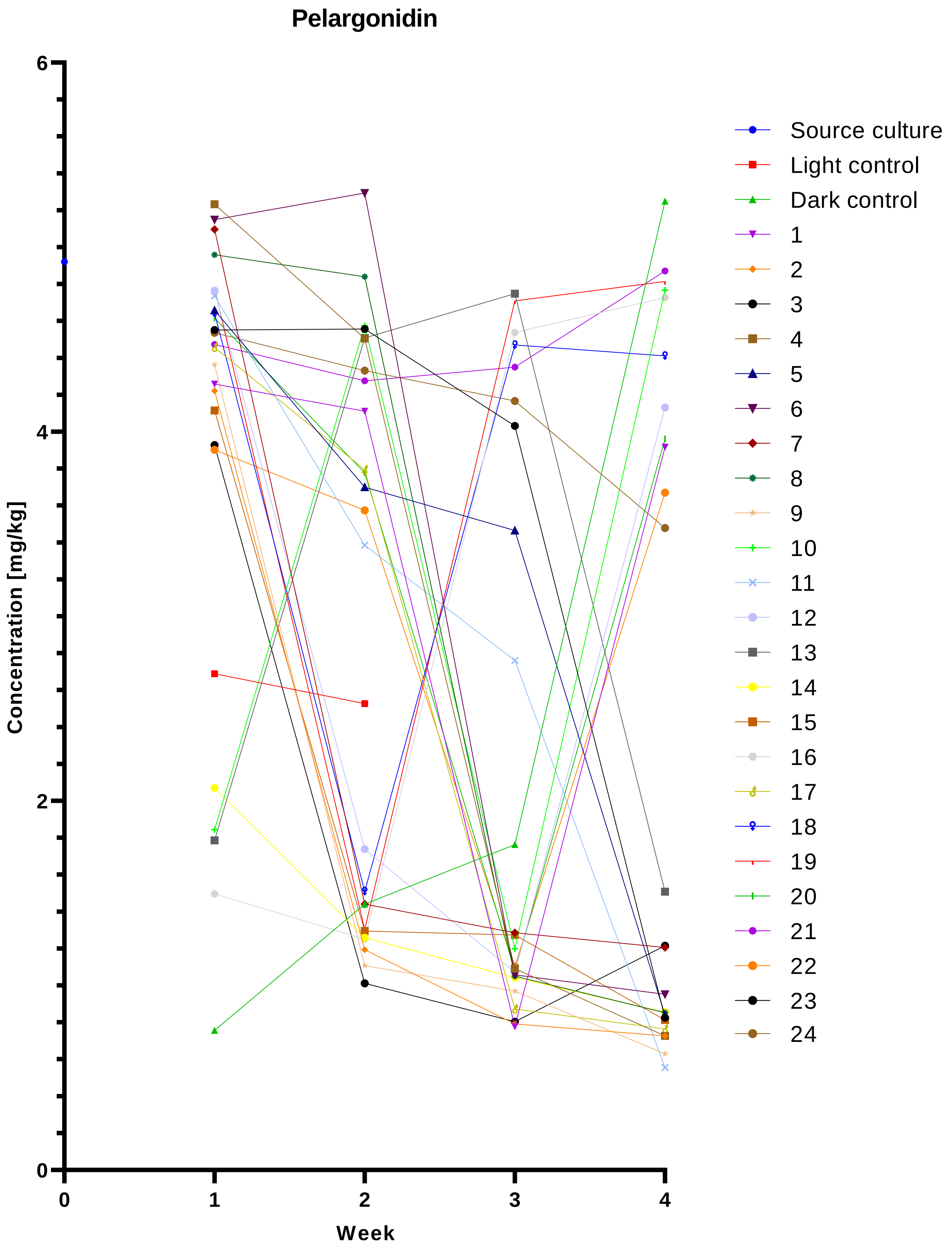

Supplementary Figure 19

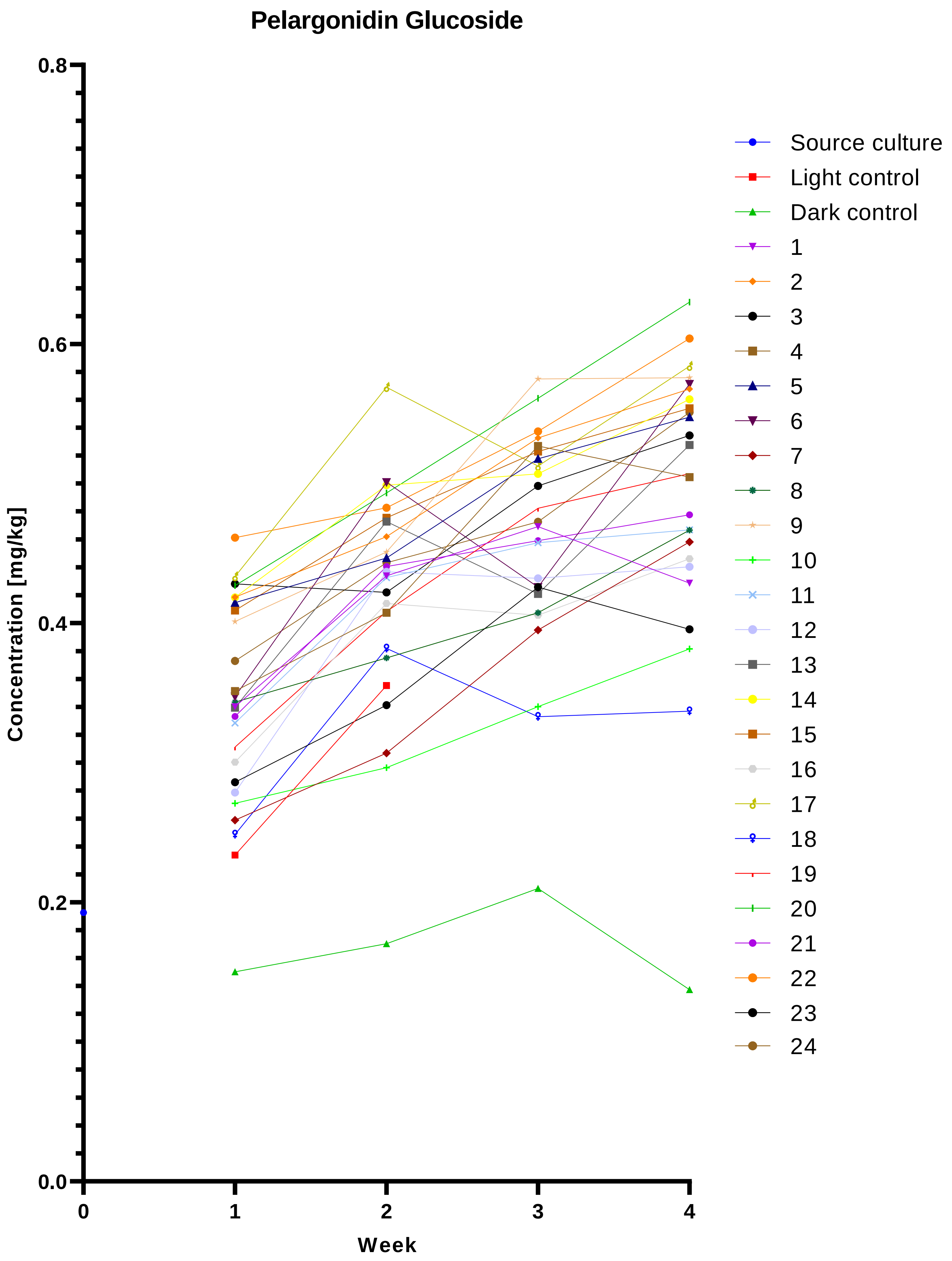

Supplementary Figure 20

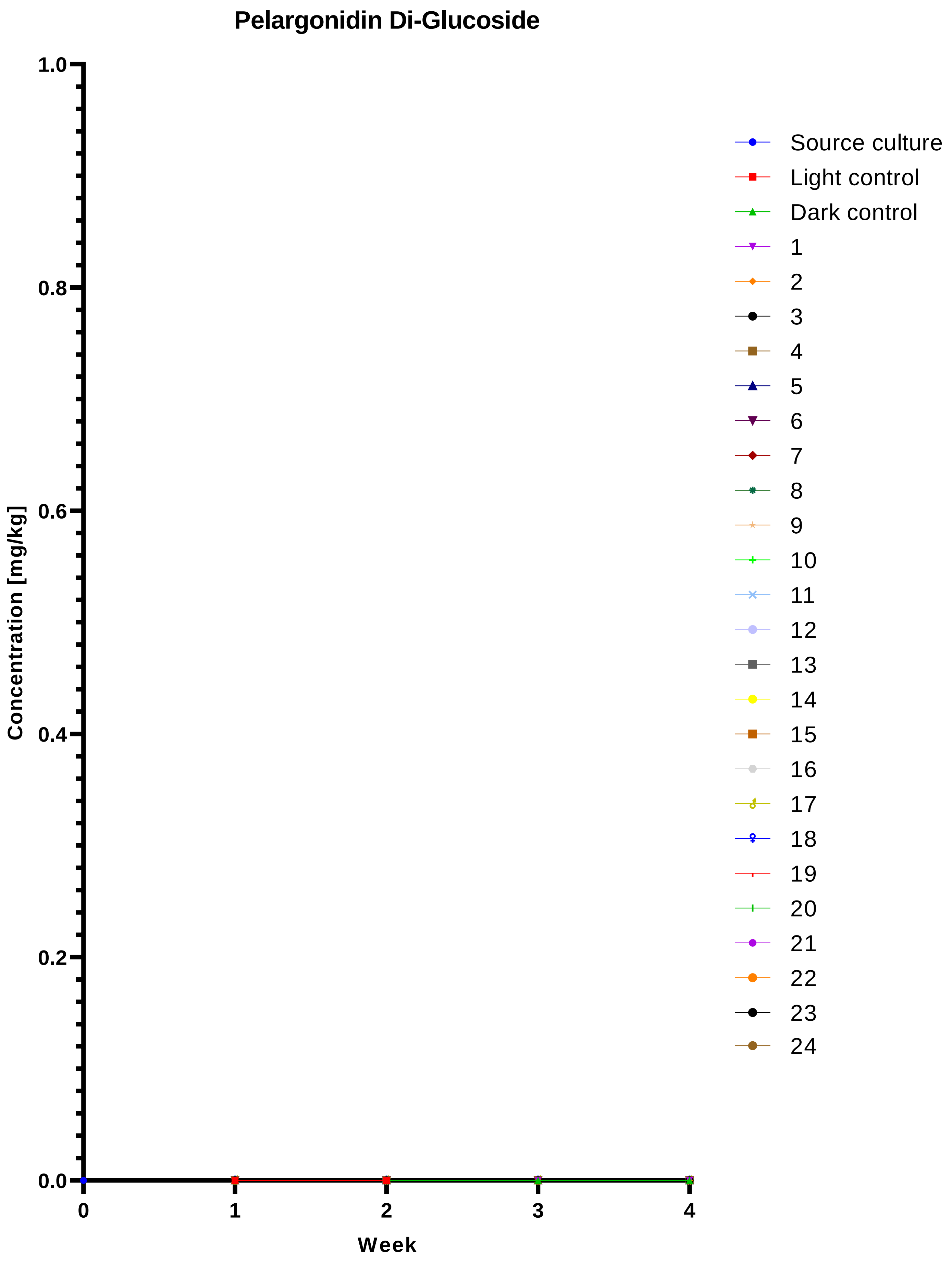

Supplementary Figure 21

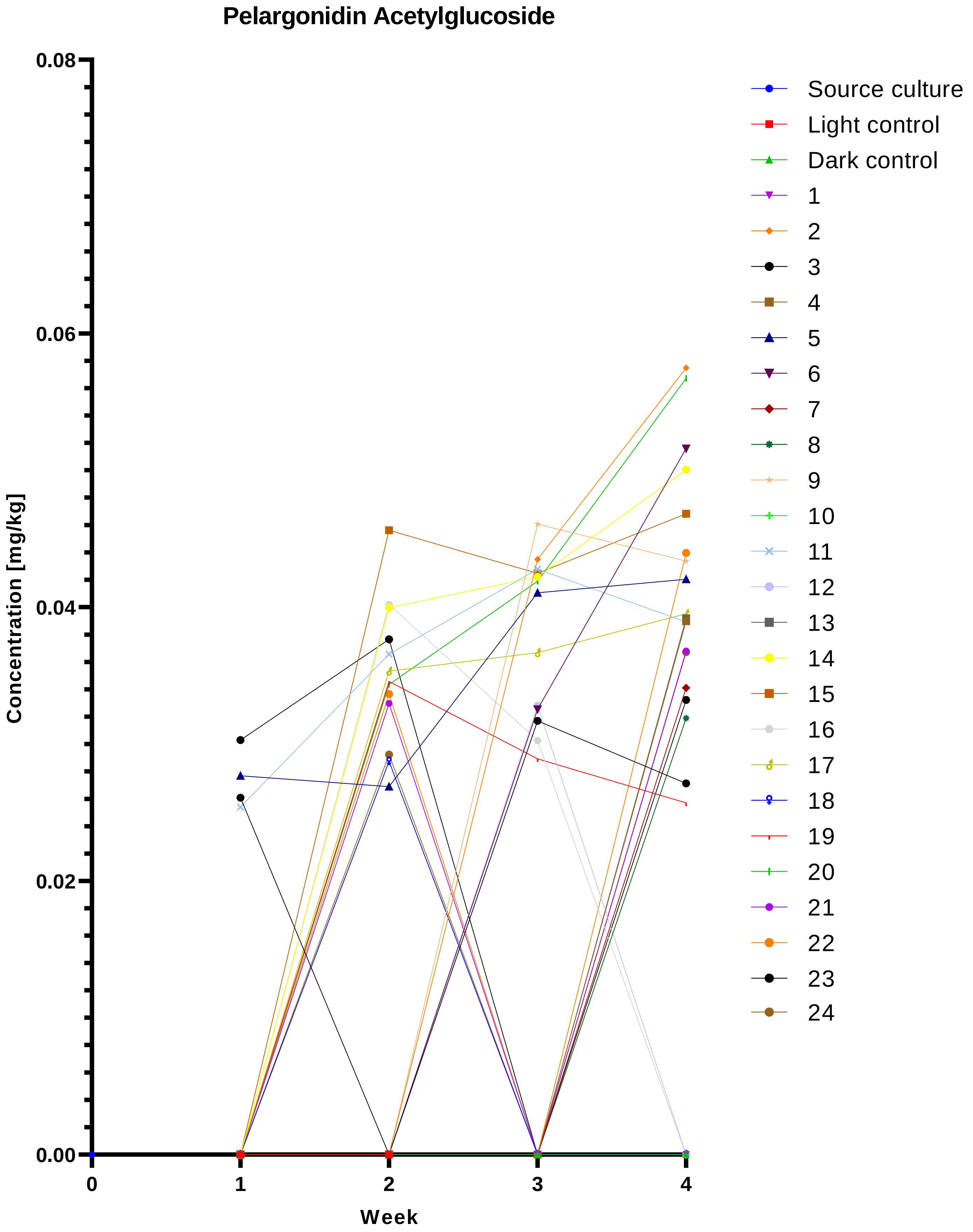

Supplementary Figure 22

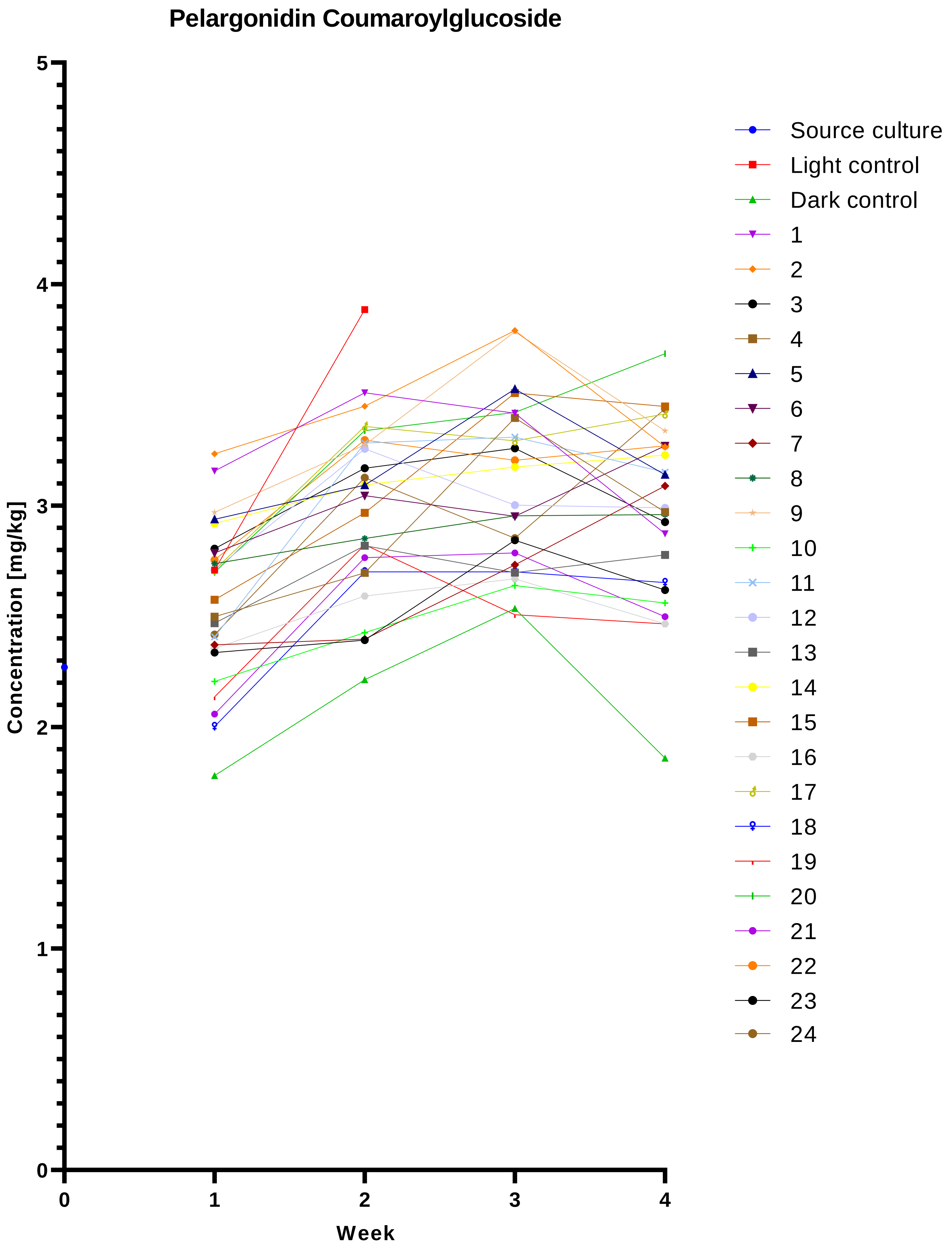

Supplementary Figure 23

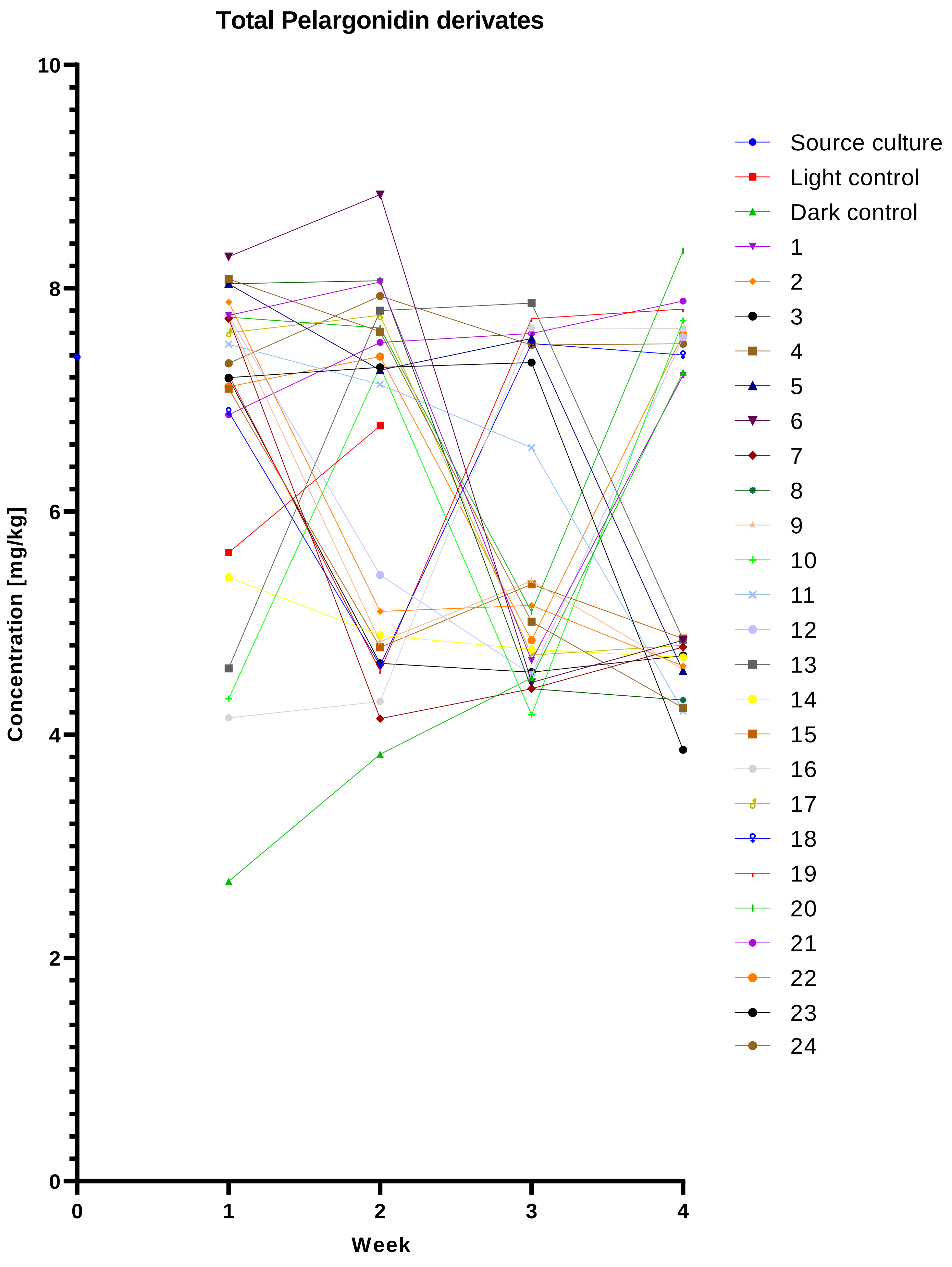

Supplementary Figure 24

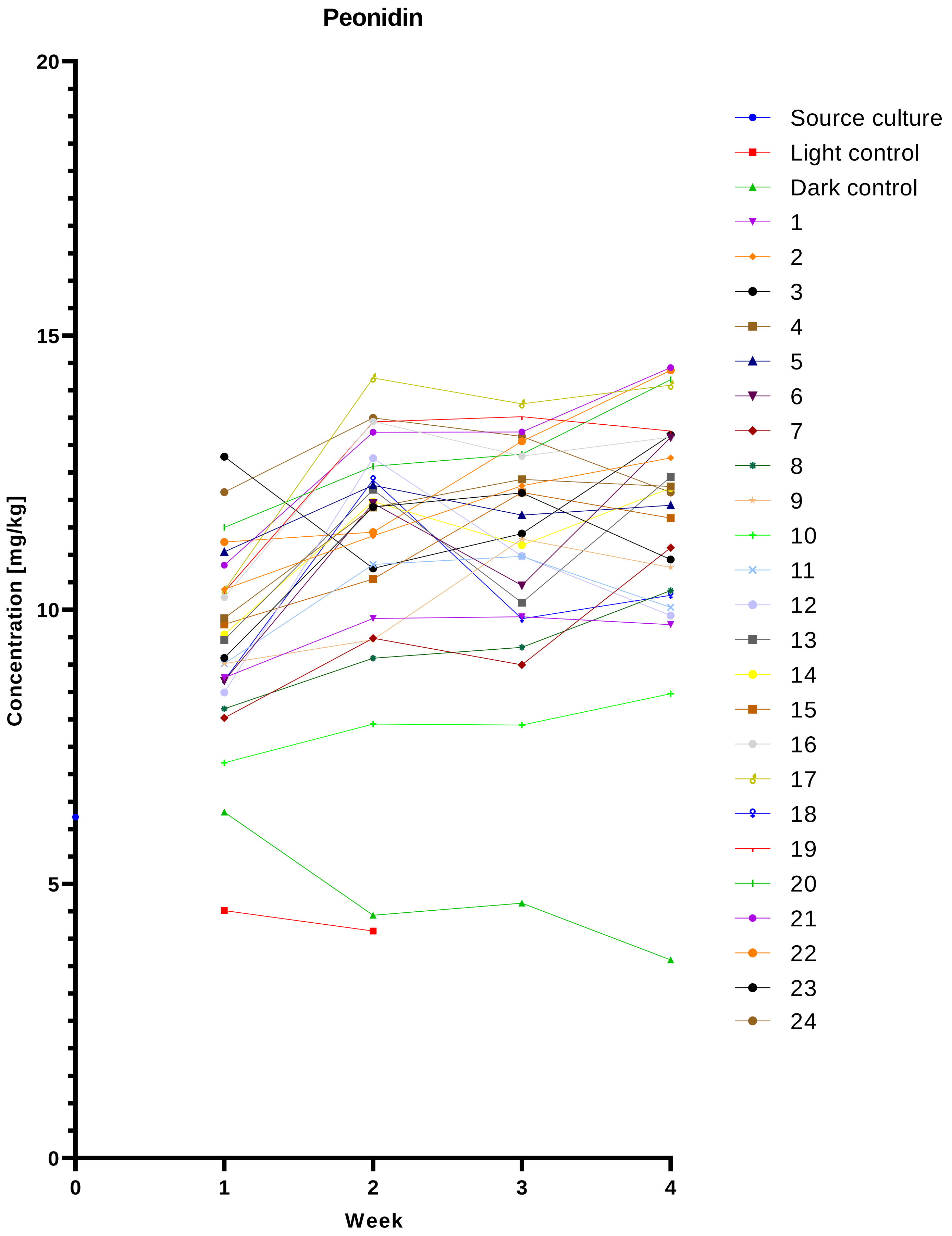

Supplementary Figure 25

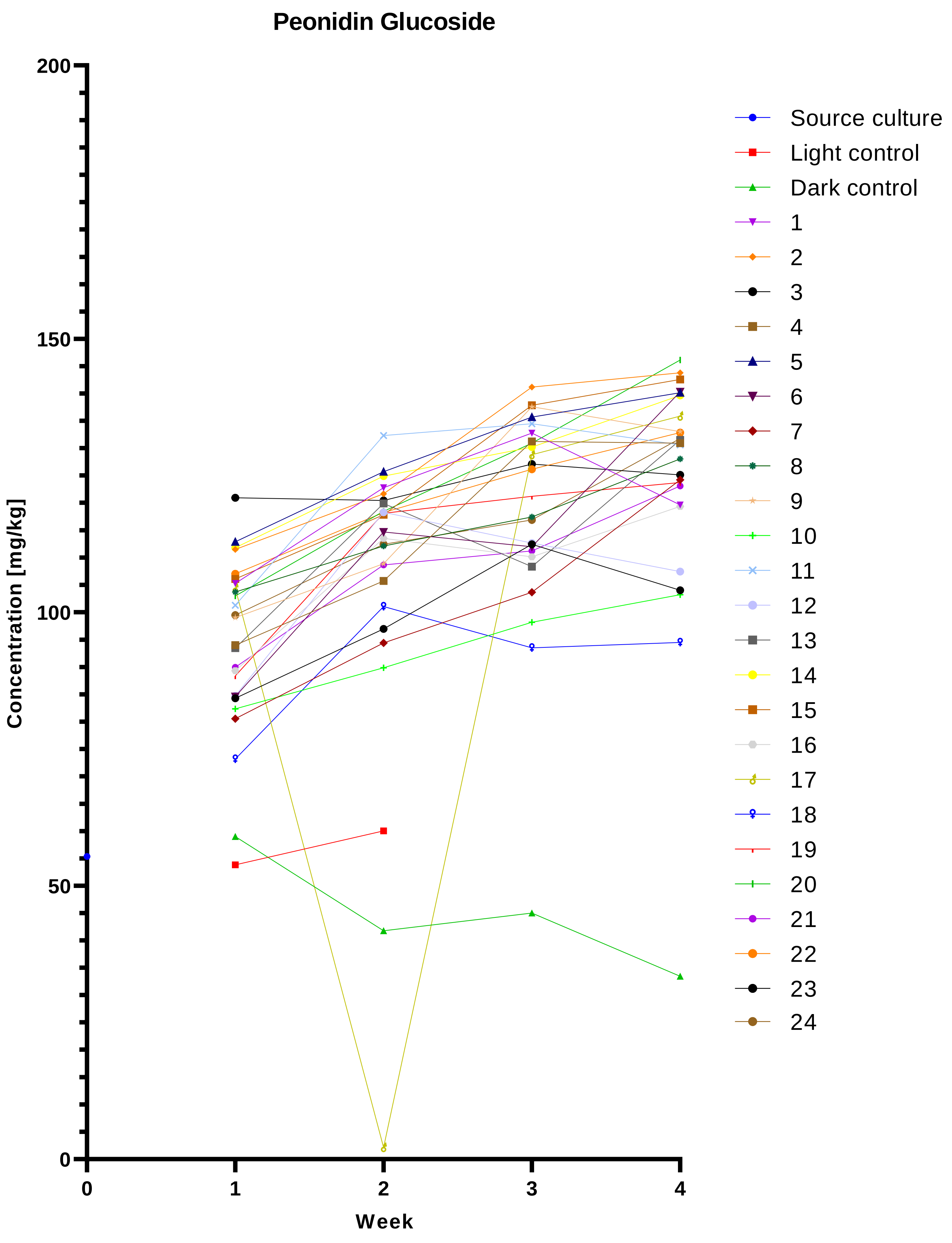

Supplementary Figure 26

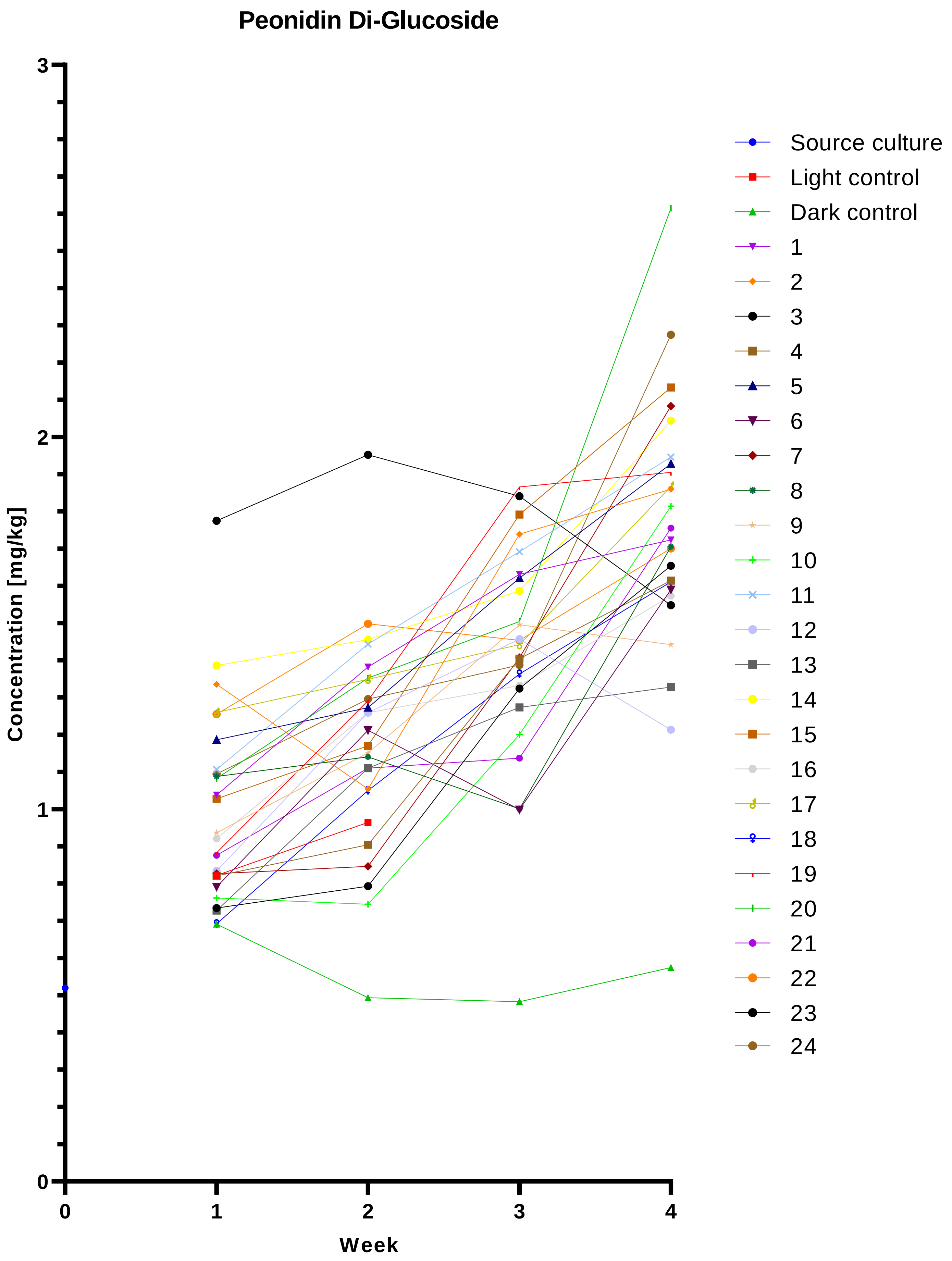

Supplementary Figure 27

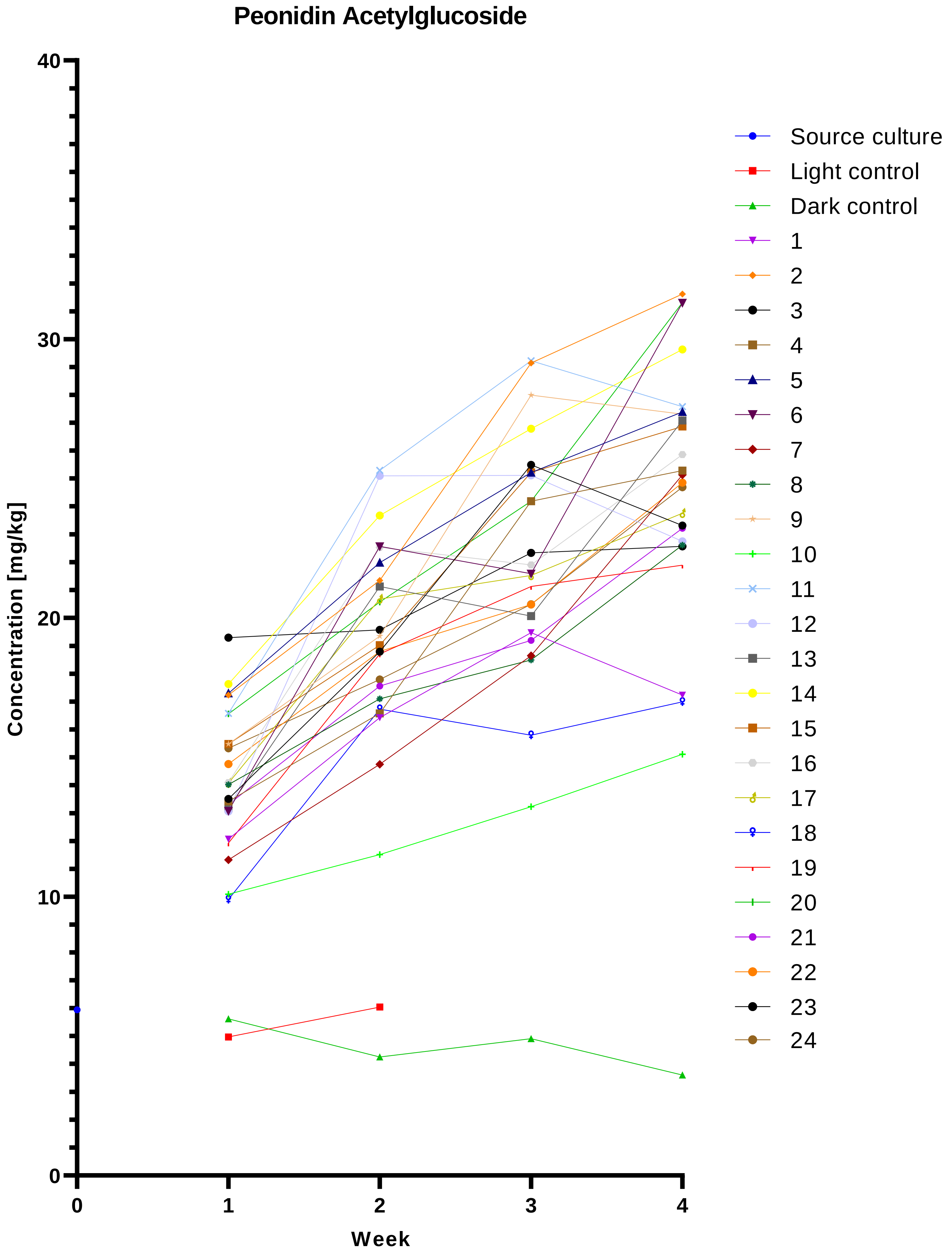

Supplementary Figure 28

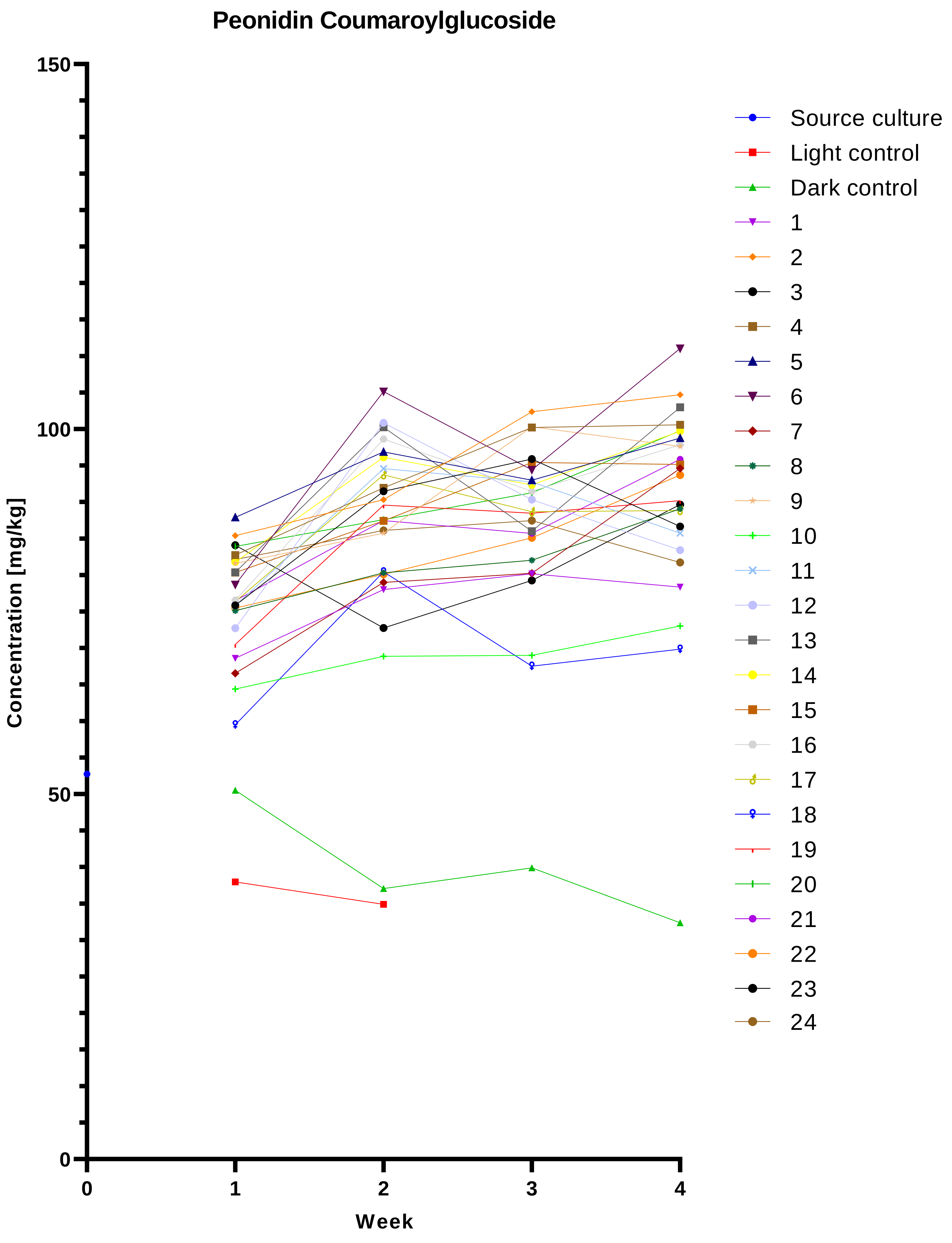

Supplementary Figure 29

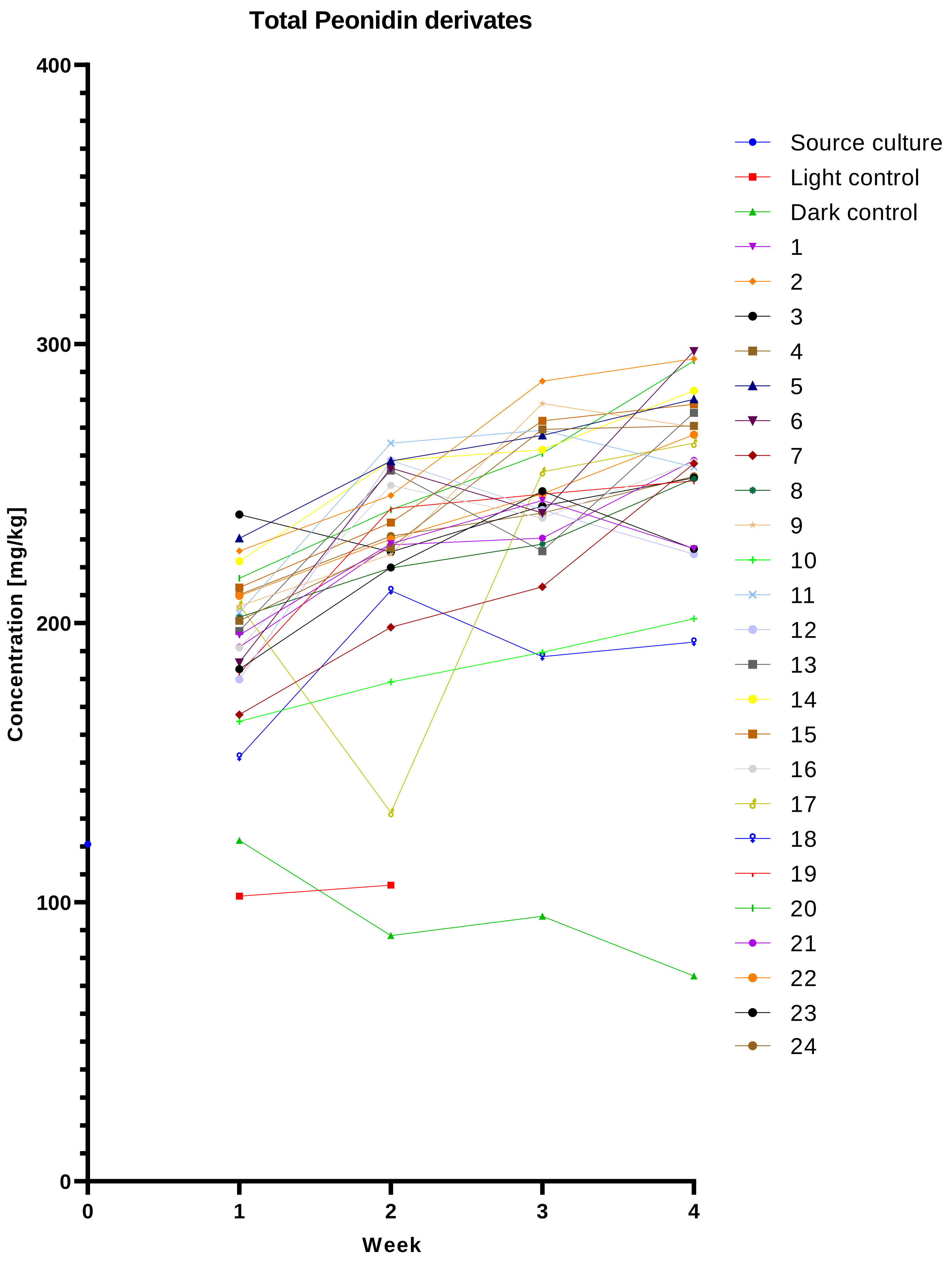

Supplementary Figure 30

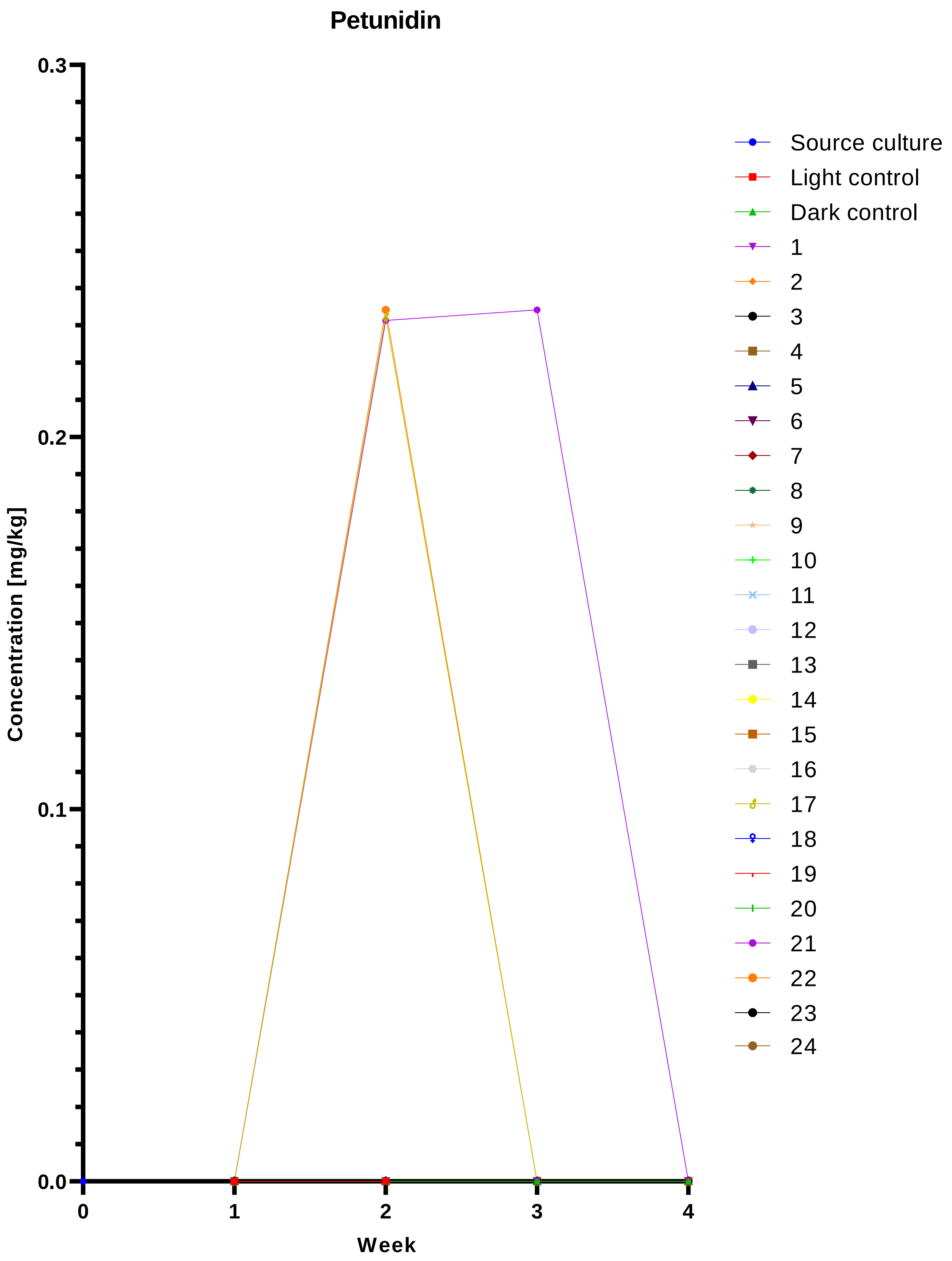

Supplementary Figure 31

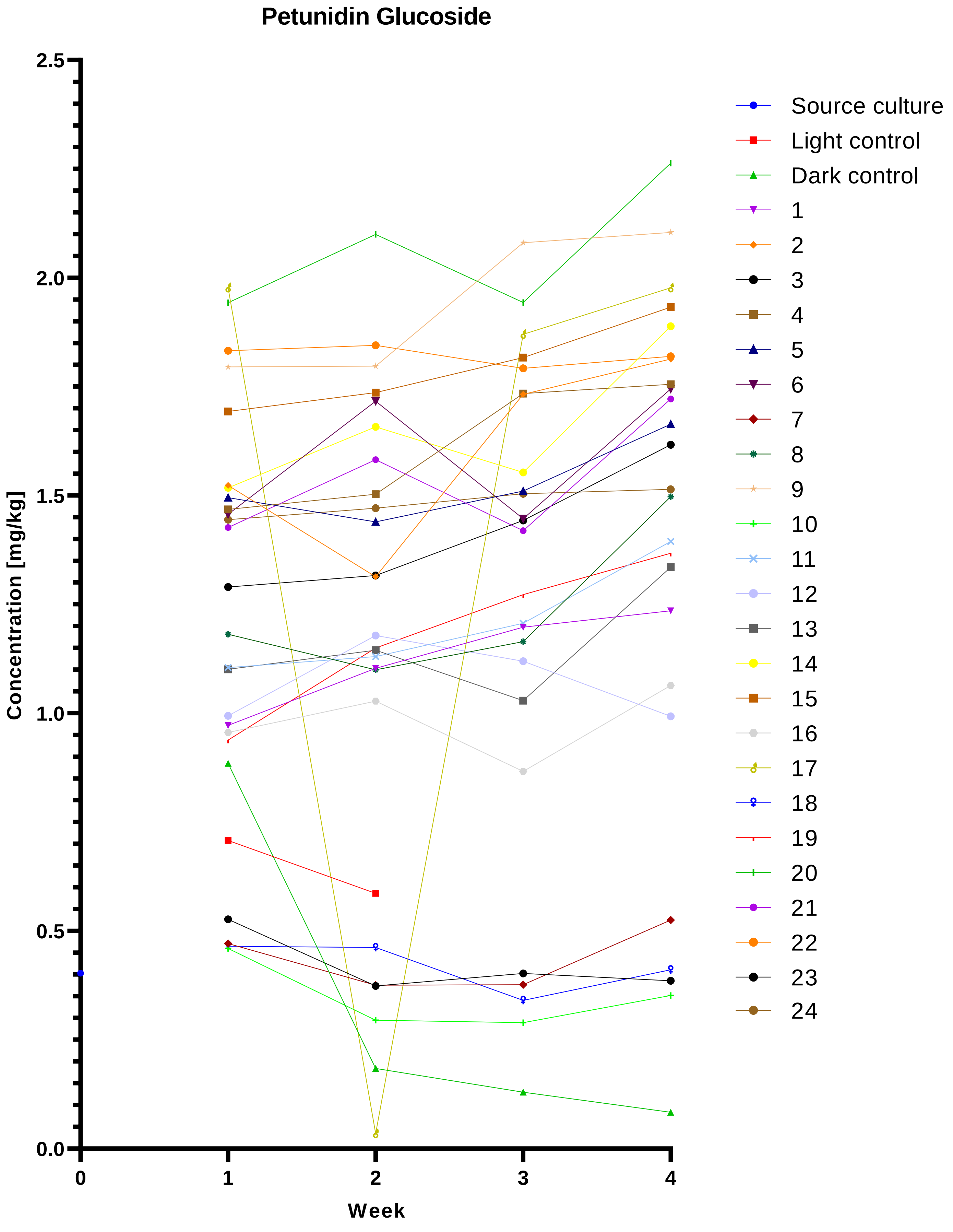

Supplementary Figure 32

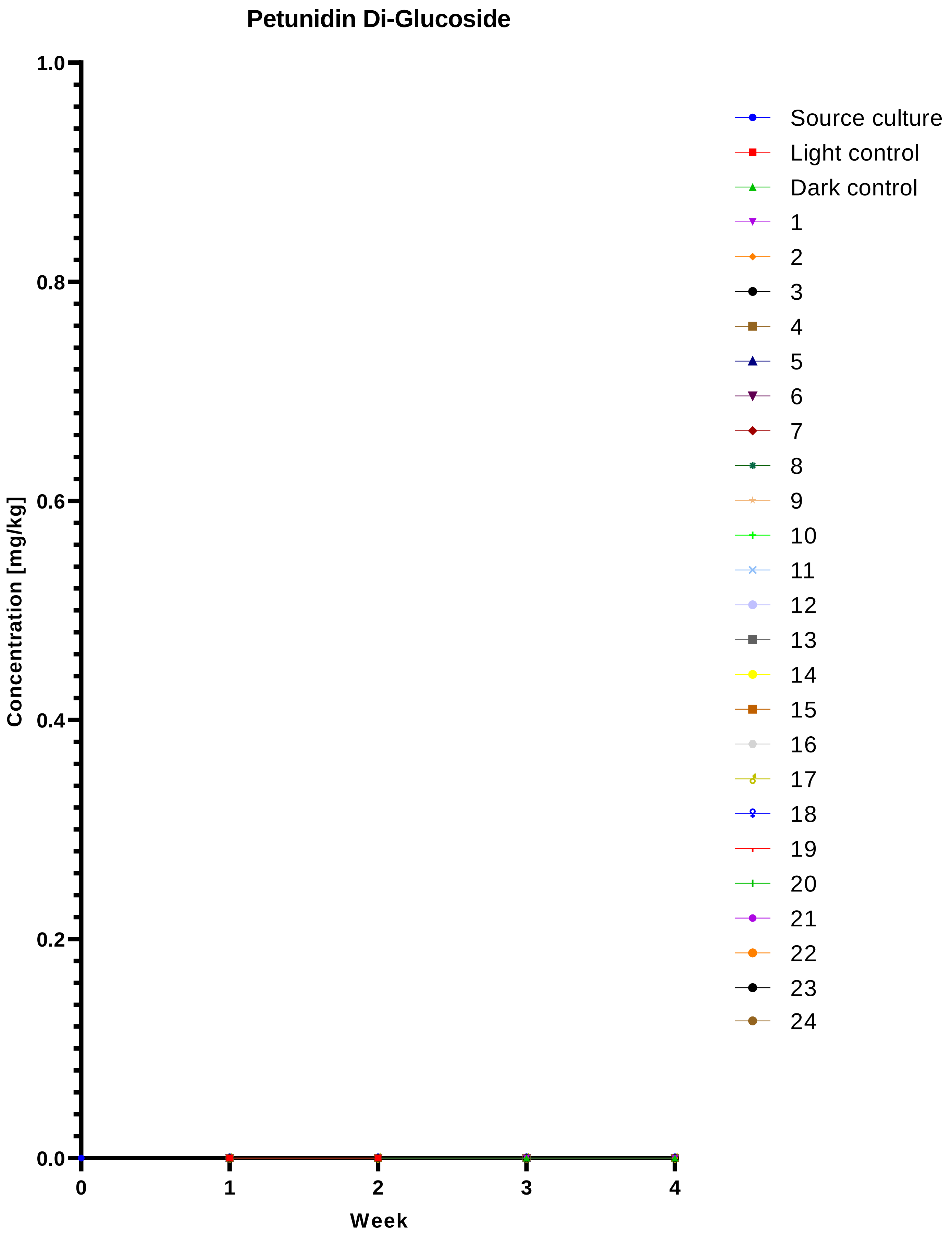

Supplementary Figure 33

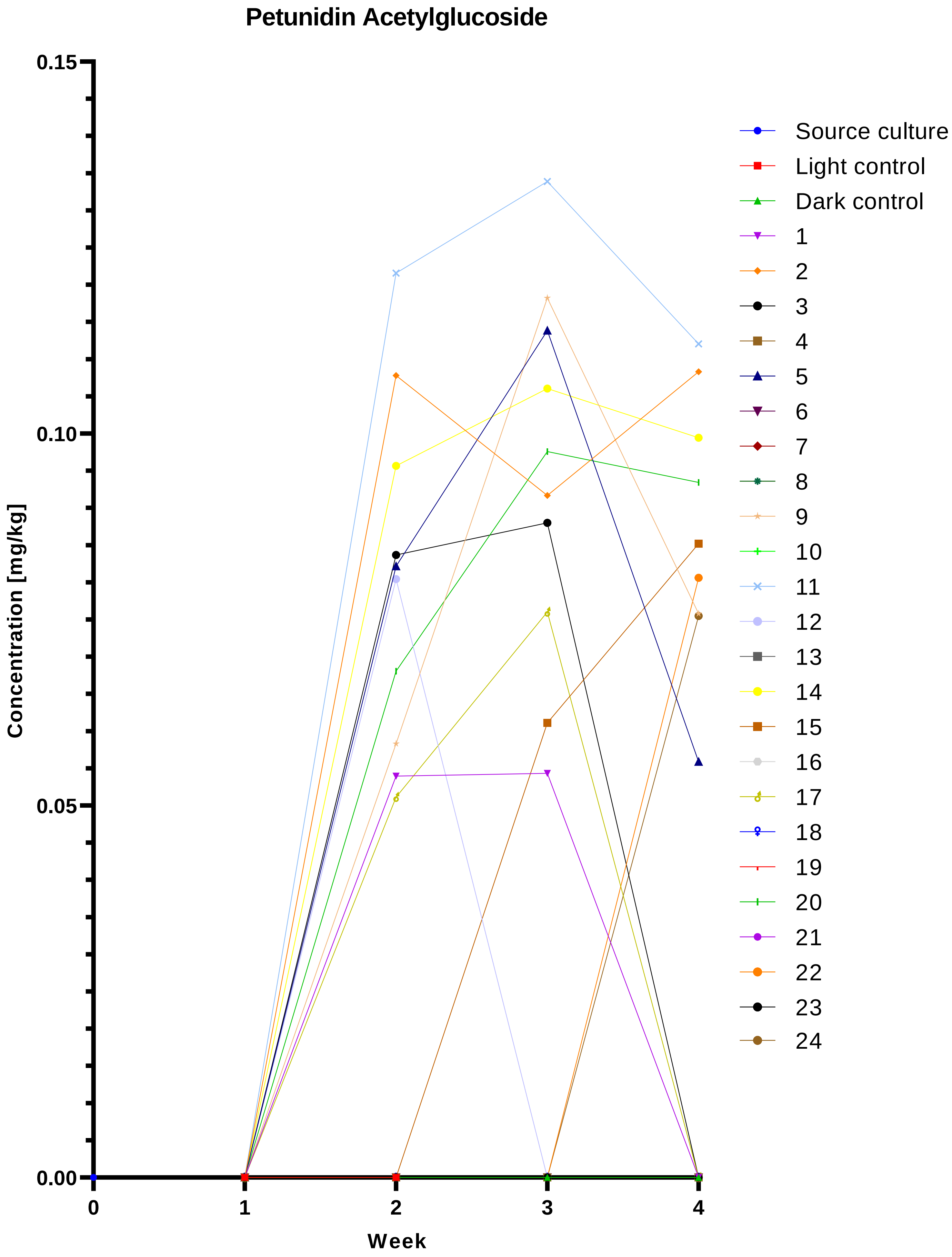

Supplementary Figure 34

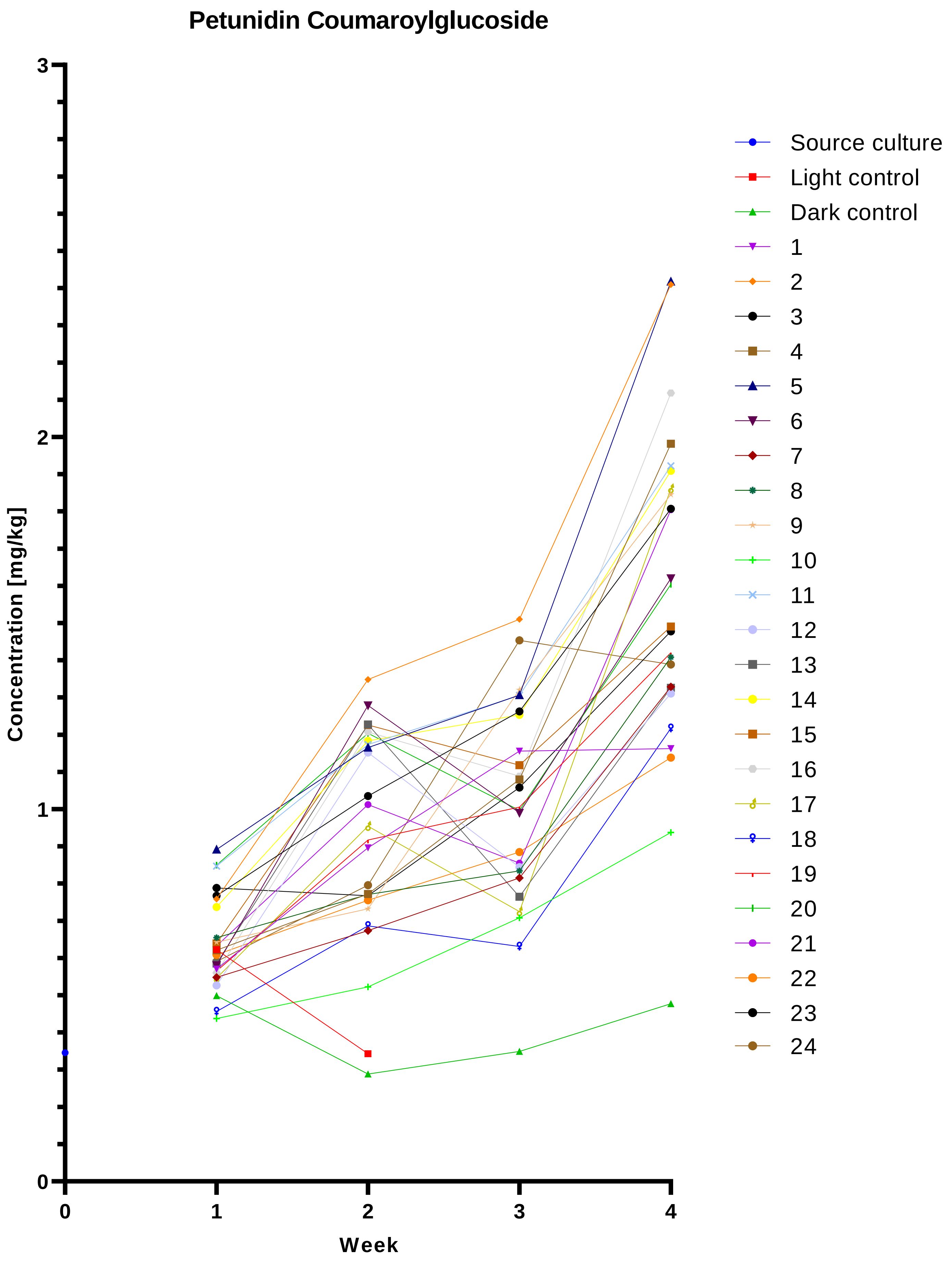

Supplementary Figure 35

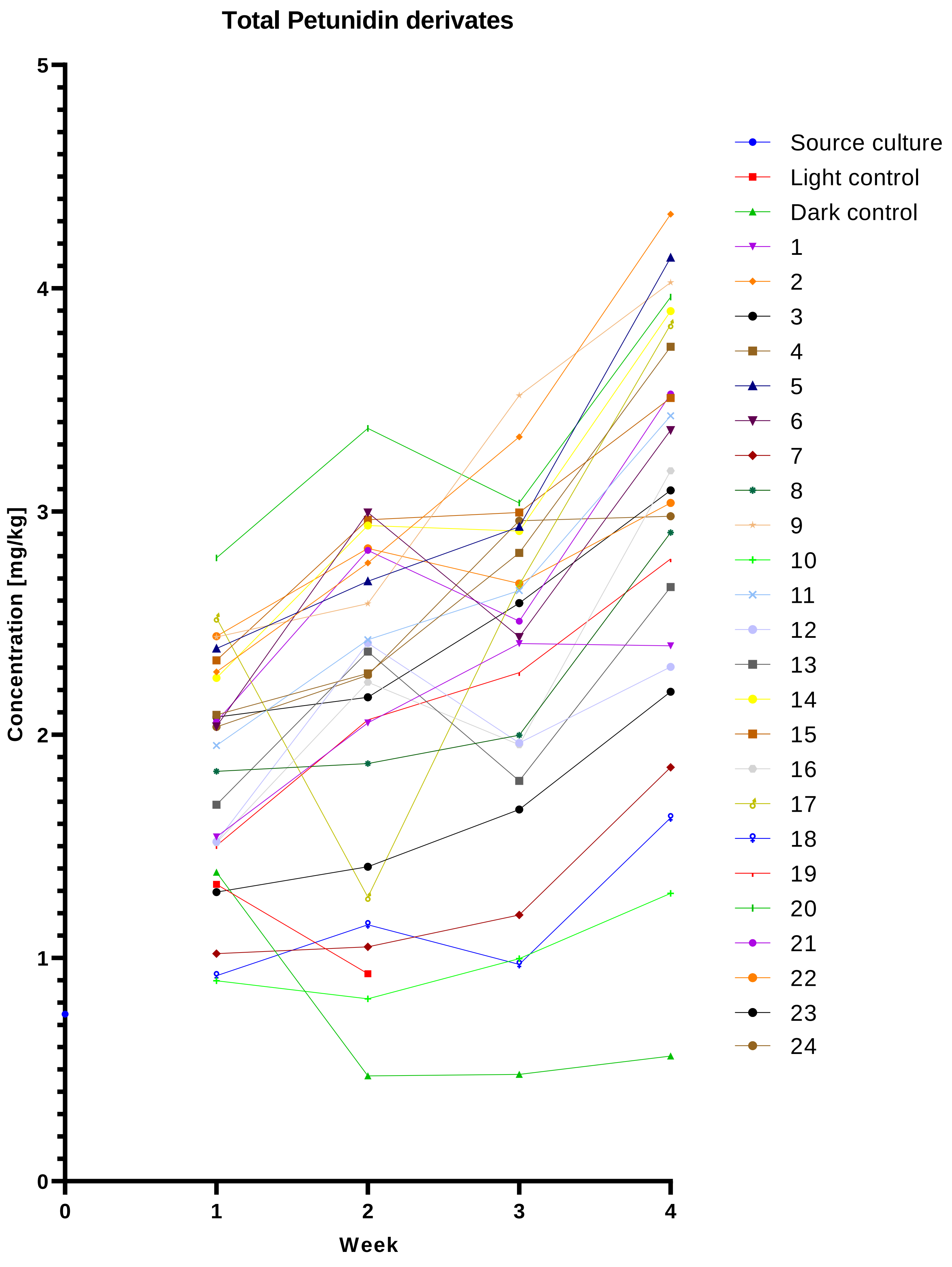

Supplementary Figure 36
